# A transposon insertion in the promoter of *BcCPR5* impairs trichome formation in wucai (*Brassica campestris* L.)

**DOI:** 10.1093/hr/uhag184

**Published:** 2026-05-08

**Authors:** Xun Gao, Jian Wang, Wenying Zhang, Kangkang Wang, Jianqiang Wu, Xiaoyan Tang, Jinfeng Hou, Guohu Chen, Chenggang Wang, Lingyun Yuan, Wenjie Wang

**Affiliations:** Anhui Provincial Engineering Research Center of Horticultural Crop Breeding, School of Horticulture, Anhui Agricultural University, Hefei 230036, China; Anhui Province Key Laboratory of Horticultural Crop Quality Biology, School of Horticulture, Anhui Agricultural University, Hefei 230036, China; Anhui Provincial Engineering Research Center of Horticultural Crop Breeding, School of Horticulture, Anhui Agricultural University, Hefei 230036, China; Anhui Province Key Laboratory of Horticultural Crop Quality Biology, School of Horticulture, Anhui Agricultural University, Hefei 230036, China; Anhui Provincial Engineering Research Center of Horticultural Crop Breeding, School of Horticulture, Anhui Agricultural University, Hefei 230036, China; Anhui Province Key Laboratory of Horticultural Crop Quality Biology, School of Horticulture, Anhui Agricultural University, Hefei 230036, China; Anhui Provincial Engineering Research Center of Horticultural Crop Breeding, School of Horticulture, Anhui Agricultural University, Hefei 230036, China; Anhui Province Key Laboratory of Horticultural Crop Quality Biology, School of Horticulture, Anhui Agricultural University, Hefei 230036, China; Anhui Provincial Engineering Research Center of Horticultural Crop Breeding, School of Horticulture, Anhui Agricultural University, Hefei 230036, China; Anhui Province Key Laboratory of Horticultural Crop Quality Biology, School of Horticulture, Anhui Agricultural University, Hefei 230036, China; Department of Vegetable Culture and Breeding, Wanjiang Vegetable Industrial Technology Institute, Maanshan 238200, China; Anhui Provincial Engineering Research Center of Horticultural Crop Breeding, School of Horticulture, Anhui Agricultural University, Hefei 230036, China; Anhui Province Key Laboratory of Horticultural Crop Quality Biology, School of Horticulture, Anhui Agricultural University, Hefei 230036, China; Department of Vegetable Culture and Breeding, Wanjiang Vegetable Industrial Technology Institute, Maanshan 238200, China; Anhui Provincial Engineering Research Center of Horticultural Crop Breeding, School of Horticulture, Anhui Agricultural University, Hefei 230036, China; Anhui Province Key Laboratory of Horticultural Crop Quality Biology, School of Horticulture, Anhui Agricultural University, Hefei 230036, China; Department of Vegetable Culture and Breeding, Wanjiang Vegetable Industrial Technology Institute, Maanshan 238200, China; Anhui Provincial Engineering Research Center of Horticultural Crop Breeding, School of Horticulture, Anhui Agricultural University, Hefei 230036, China; Anhui Province Key Laboratory of Horticultural Crop Quality Biology, School of Horticulture, Anhui Agricultural University, Hefei 230036, China; Department of Vegetable Culture and Breeding, Wanjiang Vegetable Industrial Technology Institute, Maanshan 238200, China; Anhui Provincial Engineering Research Center of Horticultural Crop Breeding, School of Horticulture, Anhui Agricultural University, Hefei 230036, China; Anhui Province Key Laboratory of Horticultural Crop Quality Biology, School of Horticulture, Anhui Agricultural University, Hefei 230036, China; Department of Vegetable Culture and Breeding, Wanjiang Vegetable Industrial Technology Institute, Maanshan 238200, China; Anhui Provincial Engineering Research Center of Horticultural Crop Breeding, School of Horticulture, Anhui Agricultural University, Hefei 230036, China; Anhui Province Key Laboratory of Horticultural Crop Quality Biology, School of Horticulture, Anhui Agricultural University, Hefei 230036, China; Department of Vegetable Culture and Breeding, Wanjiang Vegetable Industrial Technology Institute, Maanshan 238200, China; Anhui Provincial Engineering Research Center of Horticultural Crop Breeding, School of Horticulture, Anhui Agricultural University, Hefei 230036, China; Anhui Province Key Laboratory of Horticultural Crop Quality Biology, School of Horticulture, Anhui Agricultural University, Hefei 230036, China; Department of Vegetable Culture and Breeding, Wanjiang Vegetable Industrial Technology Institute, Maanshan 238200, China

## Abstract

Trichomes, specialized epidermal cells on plant leaves, contribute to resistance against both abiotic and biotic stress. Wucai, an important leafy vegetable in China, is predominantly characterized by low trichome density or even hairlessness in commercial cultivars. However, the genetic and molecular mechanisms underlying trichome loss remain largely unknown. In this study, we investigate the genetic basis of trichome loss using a high-trichome-density wucai inbred line, W7-1, and a low-trichome-density cultivar, W7-2. Through a combination of bulked segregant analysis sequencing and map-based cloning, we identified the homolog of the Arabidopsis *CPR5* (*Constitutive Pathogen Response 5*) gene located on chromosome A06 as the candidate locus. A 3365-bp transposon insertion in the promoter of W7-2 disrupts its transcriptional activity, leading to reduced expression of *BcCPR*5 and impaired trichome formation. Further analysis showed that *BcCPR5* expression is positively regulated by BcWRKY44 through a W-box cis-element in the promoter. In W7-2, this transposon insertion physically separates the W-box from the transcription start site. Population genetic analysis revealed that the insertion is prevalent among low-trichome-density or hairless cultivated varieties, reflecting selection during domestication and breeding. Collectively, our findings partially uncover the genetic mechanism underlying trichome loss in wucai and establish a novel BcWRKY44–BcCPR5 regulatory module controlling trichome formation.

## Introduction

Trichomes are specialized hair-like structures formed by the outward extension of epidermal cells in the aerial parts of plants [1]. They play diverse roles in plant growth and stress tolerance. Functioning as a physical barrier, trichomes protect against herbivore feeding [2, 3], ultraviolet (UV) radiation [4], excessive transpiration [5, 6], and exposure to toxic chemicals [7]. In addition, they contribute to water absorption and the detoxiffication of heavy metals and ozone [8–10]. In tea plants, trichomes serve as metabolic factories, accumulating characteristic compounds, such as UV-protective flavonoids, insecticidal caffeine, herbivore-deterrent volatiles, and theanine, which imparts umami flavor [11]. Furthermore, in the Venus flytrap (*Dionaea muscipula*), trichomes on the leaf surface are critical for sensing and capturing prey insects [12].

In *Arabidopsis thaliana*, numerous genes have been identified to participate in the regulation of trichome initiation, morphogenesis, and differentiation [13]. At the core of this regulatory network is a ternary MBW activator complex, composed of R2R3-MYB transcription factors [e.g. GL1 (GLABRA1), MYB23, and MYB82], the WD40-repeat protein TTG1 (TRANSPARENT TESTA GLABRA1), and bHLH factors [e.g. GL3 (GLABRA3), EGL3 (ENHANCER OF GLABRA3), and TT8 (TRANSPARENT TESTA 8)] [14]. This complex directly activates downstream genes, such as *GL2*/*TTG2* [15], which encodes a homeodomain-leucine zipper transcription factor essential for the differentiation of multiple epidermal cell types. Conversely, trichome development is negatively regulated by R3-MYB-type transcription factors, including CPC (CAPRICE), TRY (TRIPTYCHON), ETC1 (ENHANCER OF TRY AND CPC 1), ETC2 (ENHANCER OF TRY AND CPC 2), ETC3 (ENHANCER OF TRY AND CPC 3), and TCL1 (TRICHOMELESS1) [16–18]. These repressors compete with R2R3-MYB proteins for binding to bHLH factors, thereby disrupting MBW activator complex assembly. This suppression mechanism fine-tunes trichome initiation and ensures proper spatial patterning of trichome formation [15].

In *Brassica* crops, trichomes serve as an important trait capable of producing various secondary metabolites—including terpenes, acylsugars, and flavonoids—that play critical roles in pest and disease resistance [19]. So far, several genes involved in trichome differentiation and development have also been identified. Using a core collection of 290 *Brassica napus* accessions, Xuan et al. performed a genome-wide association study on leaf hair traits and revealed that differential expression of *BnaA.GL1.a*, *BnaC.SWEET4.a*, *BnaC.WAT1.a*, and *BnaC.WAT1.b* contributes to the natural phenotypic variation in trichome density [20]. Overexpression of the *TTG2* homolog *BnaA.TTG2.a.1* significantly increased trichome density in *B. napus* [21]. Ectopic expression of trichome regulatory genes from *Brassica villosa*, including *BvGL2*, *BvEGL3*, *BvTTG1*, and *BvTRY*, altered trichome formation in *Arabidopsis* [22]. It has also been proposed that a mutation in *BoTRY* underlies the dense trichome phenotype observed in *Brassica incana* [23]. In *Brassica rapa*, *BrpHL1a*, a homolog of *GL1*, was identified as the major gene regulating trichome formation on both leaf surfaces and margins, while *BrpHL1b* primarily functions at the leaf margins [24]. A loss-of-function mutation in *BrGL1* leads to glabrous leaves in Chinese cabbage [25] and *Brassica juncea* [26]. Additionally, knockout of *BrrTCP4b*, a homolog of *TEOSINTE BRANCHED1/CYCLOIDEA/PCF 4* (*TCP4*), resulted in a significant increase in trichome density in turnip (*B. rapa* var. *rapa*) [27]. Collectively, these findings demonstrate broad conservation of the trichome regulatory network between *Arabidopsis* and *Brassica* species.

Wucai (*Brassica campestris* L.), a subspecies of non-heading Chinese cabbage, is widely cultivated in southern China. It is rich in essential nutrients, including glucosinolates, vitamins [28], and free amino acids [29], which are vital for dietary diversity and human health. Notably, wucai typically exhibits low trichome density or is glabrous. However, the genetic mechanisms underlying trichome loss in wucai remain unclear, limiting the effective utilization of this trait in breeding programs. In this study, we generated a genetic population by crossing two inbred lines: the high-trichome-density line W7-1 and the low-trichome-density line W7-2. Using bulked segregant analysis sequencing (BSA-seq) combined with map-based cloning, we identified *BcCPR5* as the key gene regulating trichome density. Subsequent analyses revealed that BcWRKY44 activates the transcription of *BcCPR5* by directly binding to a W-box cis-element in its promoter. In the low-trichome-density line W7-2, however, a 3365-bp transposon insertion near this element attenuates transcriptional activation, leading to impaired *BcCPR5* expression. Population genetic analysis of natural wucai accessions further indicated that this insertion is strongly associated with glabrous phenotypes. Our findings provide valuable insights for breeding novel wucai varieties with improved stress resistance and quality traits.

## Results

### Morphology and genetics of trichome development in wucai

**Figure 1:**
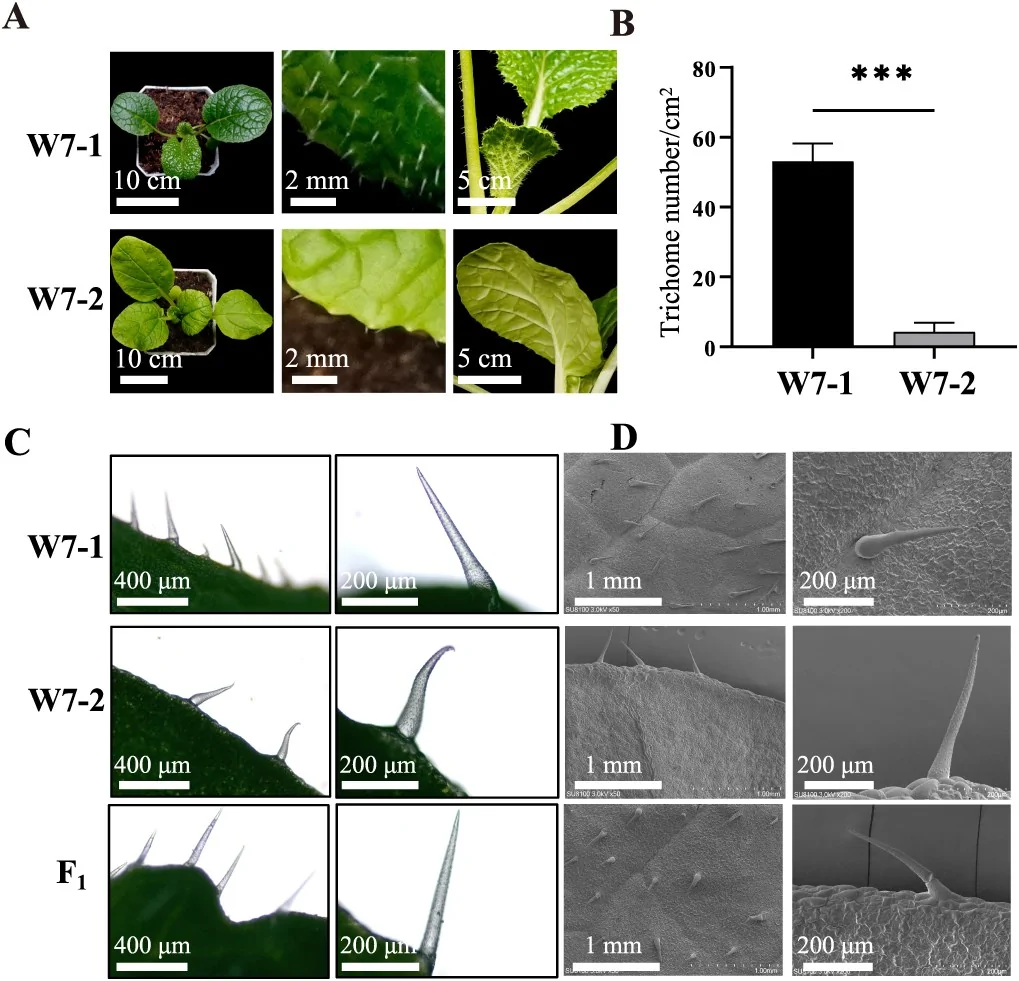
Comparison of trichome distribution and morphology among W7-1, W7-2, and F_1_ plants. (A) Macroscopic images showing the phenotype and trichome distribution in W7-1 and W7-2. (B) Quantitative analysis of leaf trichome density. Data are presented as mean ± SD (*n* = 10). Statistical significance was determined by Student’s *t*-test (^***^P < 0.001). (C) Light microscopy images of trichome structures in W7-1, W7-2, and F_1_ plants. (D) SEM images of trichome structures in W7-1, W7-2, and F_1_ plants.

Comparative analysis of trichome density in different tissues of the inbred lines W7-1 and W7-2 revealed distinct patterns of trichome distribution. In W7-1, trichomes were densely distributed on the leaves, leaf veins, and petioles. However, in W7-2, trichomes were largely restricted to the leaf surface, primarily along the margins (Fig. 1A). Quantitative assessment showed that the leaf trichome density in W7-2 was only 8% of that observed in W7-1 (Fig. 1B).

To characterize trichome morphology, we examined W7-1 and W7-2 plants using stereomicroscopy and scanning electron microscopy (SEM). The results indicated that trichomes in W7-1 are nonglandular, unicellular, and unbranched, exhibiting a distinct spine-like morphology (Fig. 1C and D), consistent with a previous report [24]. Although trichome density was significantly reduced in W7-2, no obvious morphological defects were observed (Fig. 1C and D).

To investigate the genetic determinants of trichome density variation, a segregating population was established through a cross between W7-1 and W7-2. All F_1_ individuals displayed a high-trichome-density phenotype with normal trichome morphology (Fig. 1C and D), indicating complete dominance of the high-density trait. Genetic segregation analysis of the F_2_ population showed that the distribution of high- and low-trichome-density plants conformed to a 3:1 ratio (*χ*^2^ = 2.67, *P* > 0.05) (Table S1), consistent with monogenic Mendelian inheritance.

### Identification of *BcCPR5* as the candidate gene for trichome density variation

**Figure 2:**
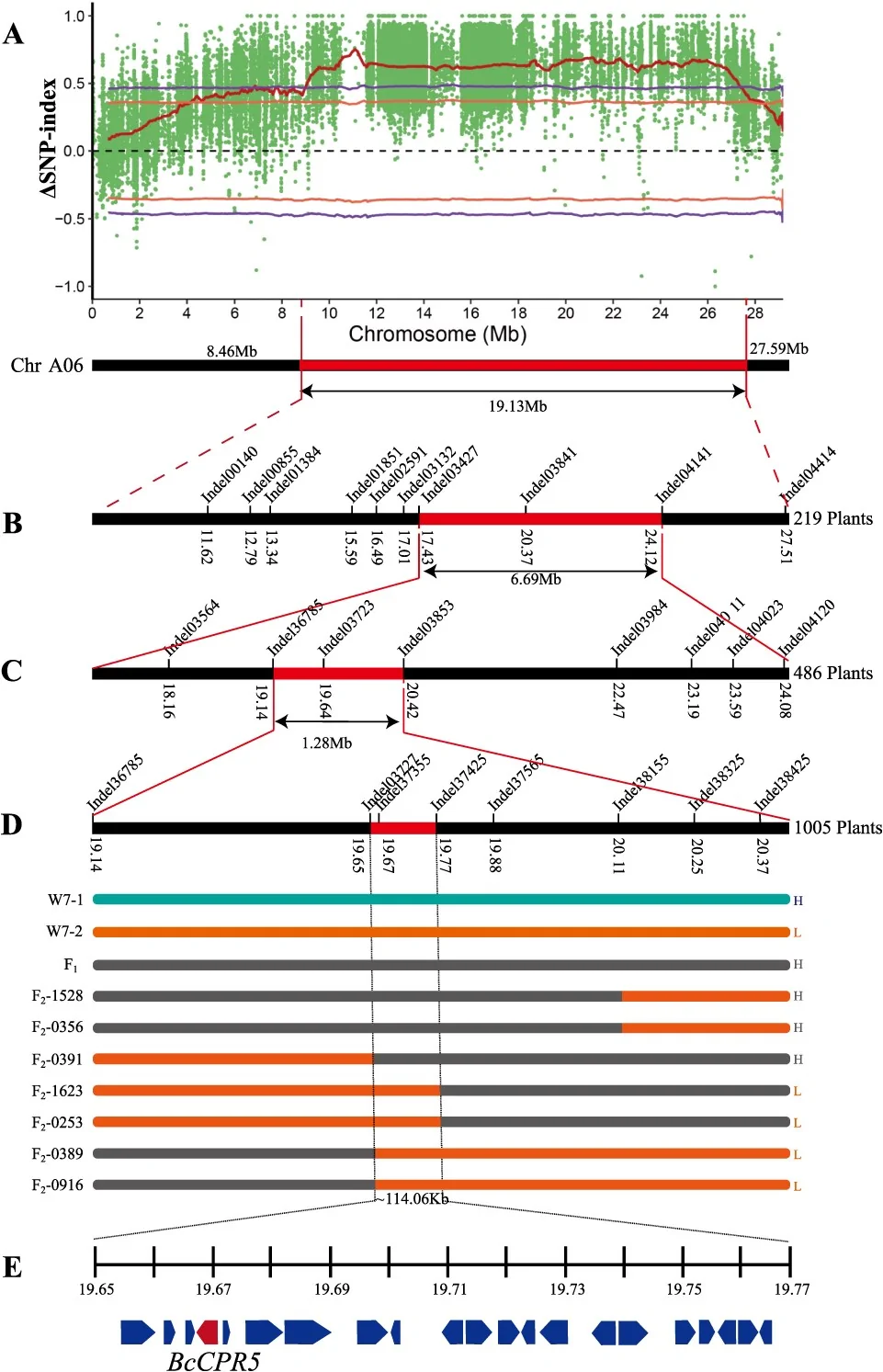
Genetic mapping of the trichome trait in wucai. (A) BSA-seq analysis identifying the candidate genomic region on chromosome A06. (B–D) Progressive fine-mapping using recombinant populations: (B) 219 F_2_ plants (2022 population), (C) 486 F_2_ plants (2023 spring population), (D) 1005 F_2_ plants (2023 autumn population). H, high-trichome-density phenotype; L, low-trichome-density phenotype. (E) Final candidate region containing 21 annotated genes, with *LOC103873918* (*BcCPR5*) identified as the key candidate gene.

To identify candidate gene associated with trichome density in wucai, we performed BSA-seq. Genomic DNA was pooled from 30 high-trichome-density individuals and 30 low-trichome-density F_2_ individuals, along with five plants from each parental line. Whole-genome sequencing generated 60.81 Gb of high-quality clean data. The high-trichome-density and low-trichome-density pools yielded 15.44 and 16.01 Gb of clean bases, respectively, while the parental lines W7-1 and W7-2 contributed 14.92 and 14.44 Gb (Table S2), achieving genome coverages of 33×, 34×, 32×, and 31×, respectively (Table S3). Single nucleotide polymorphism (SNP) index analysis identified a 19.13-Mb candidate region on chromosome A06 (8.46–27.59 Mb) that exceeded the 95% confidence threshold, indicating a strong association with variation in trichome density (Fig. 2A).

Using resequencing data from the parental lines, we developed 25 InDel markers evenly distributed across the candidate region. All markers exhibited consistent polymorphisms among W7-1, W7-2, and their F_1_ progeny. Based on 10 informative InDel markers spanning the candidate region, initial linkage analysis was conducted on 219 F_2_ individuals displaying the recessive low-trichome-density phenotype from the 2022 population. Twenty recombinants were identified, narrowing the target locus to a 6.69-Mb interval on chromosome A06 (17 432 300–24 120 673 bp), flanked by markers InDel03427 (four recombinants) and InDel04141 (two recombinants; Fig. 2B). Fine-mapping was subsequently performed using 486 individuals from the 2023 spring population genotyped with eight polymorphic markers. This analysis revealed 15 recombinants and further refined the candidate interval to 1.28 Mb between markers InDel36785 and InDel03853 (Fig. 2C). One more round of fine-mapping on 1005 F_2_ individuals from the 2023 autumn population, using eight additional markers, identified 29 recombinants. Phenotypic validation of recombinant F_2_ families confirmed the target trait genotype, ultimately delimiting the candidate region to a 114.06-kb interval (19 654 765–19 768 826 bp) between markers InDel03727 (three recombinants) and InDel37425 (two recombinants) (Fig. 2D).

Genome annotation of the 114.06-kb candidate region revealed 21 predicted genes (Fig. 2E and Table S4). Transcripts of eight genes were detected in leaf tissue through reverse-transcription quantitative PCR (RT-qPCR) (Fig. S1). Sequencing analysis further revealed that among these eight genes, only two, *LOC103873915* and *LOC103873918*, carry mutations in their coding regions and harbor SNPs/indels in their promoter regions between the lines W7-1 and W7-2 (Table S4). For *LOC103873915*, the SNPs in the coding region result in synonymous mutations (Table S4), and its expression level showed no significant difference between the two parental lines despite the presence of promoter polymorphisms (Fig. S1). Therefore, this gene was excluded from further analysis. In contrast, *LOC103873918* contains both large indels and SNPs in its promoter and gene body, and its expression level differed significantly between the two parents. Functional annotation indicates that *LOC103873918* encodes the homolog of the *Arabidopsis* Constitutive Expressor of Pathogenesis-Related Genes 5 (CPR5), a protein previously implicated in trichome formation and development [30–32]. Accordingly, *LOC103873918*, hereafter designated *BcCPR5*, emerged as the most promising candidate controlling trichome formation.

### BcCPR5 functions in trichome formation in wucai

To investigate the role of *BcCPR5* in trichome formation in wucai, we employed the cabbage leaf curl virus (CaLCuV)-based virus-induced gene silencing (VIGS) system [33] to silence *BcCPR5* expression in the high-trichome-density W7-1. A fragment corresponding to the predicted functional domain of BcCPR5 was cloned into the pCVA vector, generating pCVA::*BcCPR5*. As a positive control for silencing, we constructed pCVA::*BcPDS* targeting phytoene desaturase, while the empty vector (pCVA::00) served as a negative control.

**Figure 3:**
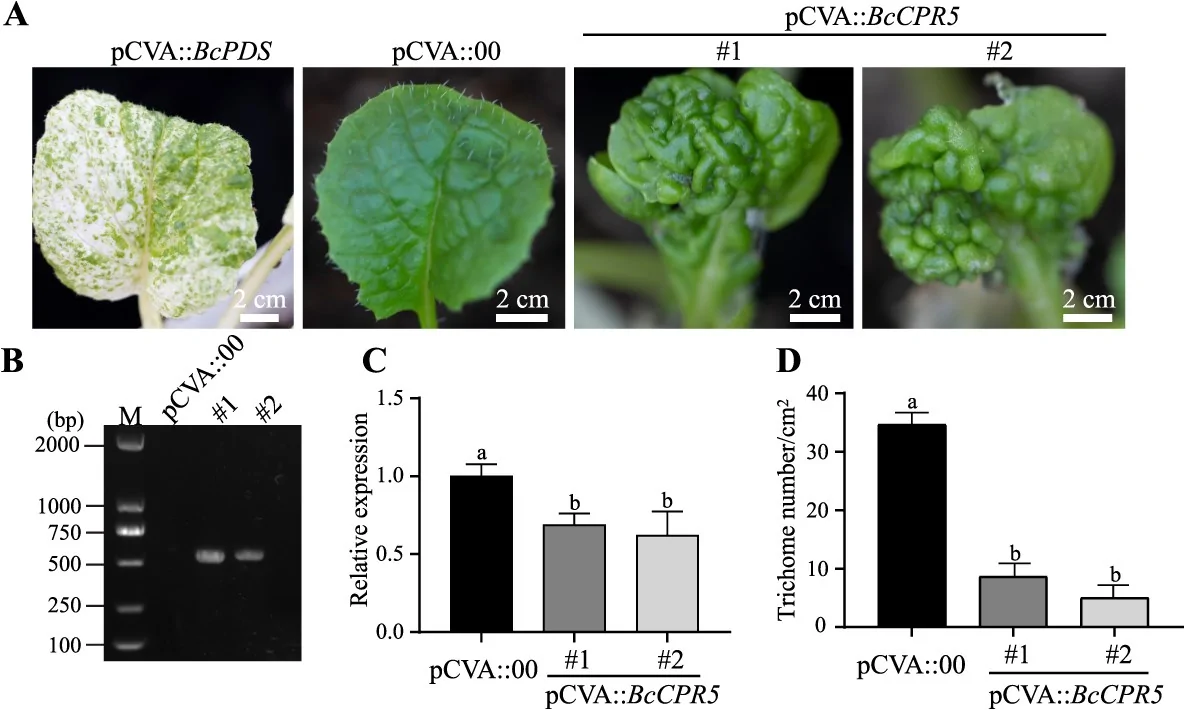
VIGS of *BcCPR5* in the W7-1 background. (A) Phenotypes of VIGS-treated plants. (B) PCR-based genotyping confirmation of unsilenced and silenced lines. (C) Relative *BcCPR5* transcript levels in unsilenced control and silenced lines, determined by qRT-PCR. (D) Trichome density in unsilenced control and silenced lines. Data are presented as mean ± SD [one-way analysis of variance (ANOVA), Tukey’s HSD test]. Different lowercase letters indicate statistically significant differences at *P* < 0.05.

Four weeks postinoculation, plants infiltrated with pCVA::*BcPDS* exhibited a typical leaf bleaching phenotype (Fig. 3A), confirming successful viral infection and efficient gene silence *via* the CaLCuV system. The presence of the viral vector was subsequently verified in pCVA::*BcCPR5*-infiltrated plants (Fig. 3B). RT-qPCR analysis revealed that *BcCPR5* transcript levels were significantly reduced in these plants compared to pCVA::00 control (Fig. 3C). Concomitantly, trichome density decreased to approximately one-sixth of that observed in control plants (Fig. 3D). Collectively, these results demonstrated that *BcCPR5* is required for trichome formation in wucai and support the mutation conclusion that a mutation at the *BcCPR5* locus is responsible for the low-trichome-density phenotype in W7-2. 

### Contribution of BcCPR5 coding-sequence alleles to trichome development

To identify the genetic variation underlying the reduced function loss of *BcCPR5* in W7-2, we compared the genomic sequences of *BcCPR5* between W7-1 and W7-2. Sequence alignment revealed numerous nucleotide polymorphisms in both exonic and intronic regions of *BcCPR5* (Table S5). Specifically, compared to W7-1, the *BcCPR5* sequence in W7-2 harbored a 43-bp deletion within the first exon, as well as eight nonsynonymous mutations and a two-amino-acid insertion in the subsequent coding sequence (CDS) (Fig. S2A). Although the deletion was predicted to introduce a frameshift mutation and a premature termination codon that would truncate 95.5% of the BcCPR5 protein (Fig. S2B), we identified a putative downstream start codon (ATG2) in frame following the deletion (Fig. S2A). This configuration could potentially initiate translation of a protein lacking the first 39 amino acids, designated *BcCPR5*^W7-2^. Multiple sequence alignment revealed that this N-terminal region is variable across species but relatively conserved within *Brassica* (Fig. S3). We therefore hypothesized that the 43-bp deletion might underlie the trichome density variation between W7-1 and W7-2.

To assess the functional consequence of these sequence differences, we generated 35S promoter-driven overexpression constructs that included the full-length *BcCPR5* from W7-1 (*35S::BcCPR5^W7-1(ATG1)^*) and the truncated form from W7-2, initiating at ATG2 (*35S::BcCPR5^W7-2(ATG2)^*). Moreover, to specifically evaluate the effects of the eight nonsynonymous mutations and the two-amino-acid insertion independent of the 43-bp deletion, we also produced a construct containing a version corresponding to the ATG2-initiated fragment but derived from the W7-1 sequence (*35S::BcCPR5*^*W7-1*(*ATG2*)^). These constructs were introduced into the Arabidopsis *cpr5* mutant for complementation assays (Fig. S4A). Genotyping confirmed successful transformation in all lines (Fig. S4B), and qRT-PCR analysis showed that *BcCPR5* transcript levels were significantly higher than the *cpr5* mutant control (Fig. S4E).

Consistent with previous reports [30], the Arabidopsis *cpr5* mutant exhibited significantly reduced trichome number, with most trichomes either unbranched or bearing only two branches (Fig. S4D). Complementation with *BcCPR5^W7-1(ATG1)^* fully rescued this phenotype, restoring both high trichome density and normal three-branched morphology (Fig. S4D). Surprisingly, ectopic expression of both *35S::BcCPR5^W7-1(ATG2)^* and *35S::BcCPR5^W7-2(ATG2)^* can also rescue the mutant phenotype to a comparable extent (Fig. S4D).

Given that the presence of ATG1 and the 43-bp deletion in the native *BcCPR5^W7-2^* genomic context might affect the utilization of ATG2 *in vivo*, we additionally transformed the full-length *BcCPR5^W7-2^* sequence, initiating from ATG1 (*BcCPR5^W7-2(ATG1)^*) into the *cpr5* mutant (Fig. S4B and C). Phenotypic analysis revealed that this construct also fully complemented the trichome defects (Fig. S4F).

Taken together, these findings demonstrate that BcCPR5 plays a conserved function analogous to *CPR5*. Neither the 43-bp deletion nor the associated amino acid substitutions and insertion alter *BcCPR5* function, suggesting that these coding-sequence variations are not responsible for the reduced trichome density observed in W7-2.

### Identification and functional analysis of a promoter insertion modulating *BcCPR5* expression

**Figure 4:**
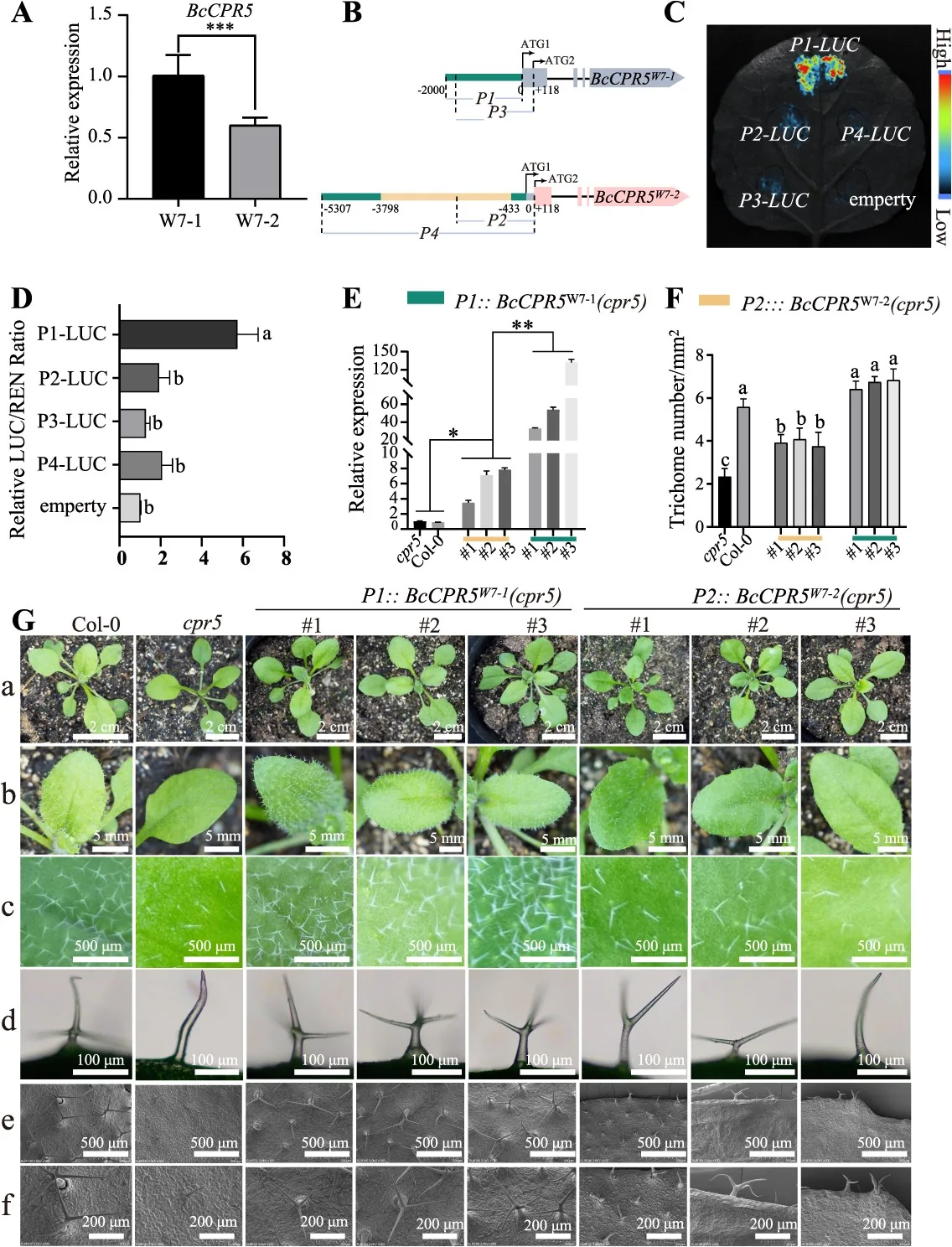
Promoter activity analysis and functional validation of BcCPR5. (A) Relative expression levels of *BcCPR5* in W7-1 and W7-2. Data are presented as mean ± SD (Student’s *t*-test, ^***^P < 0.001). (B) Schematic diagram of the promoter regions of *P1*, *P2, P3*, and *P4*. (C) Luminescence imaging of promoter activity in *N. benthamiana* leaves, indicating promoter activity for *P1*, *P2*, *P3*, and *P4*. (D) Quantitative analysis of dual-LUC activity expressed as the LUC/REN ratio. Data are presented as mean ± SD (one-way ANOVA with Tukey’s HSD test; different lowercase letters indicate significant differences at *P* < 0.05). (E) Relative expression levels of *CPR5* in wild-type (Col-0), *cpr5* mutant, and transgenic plants expressing *BcCPR5^W-1^* or *BcCPR5^W7-2^* driven by their endogenous promoters *P1* and *P2*. Data are presented as mean ± SD. Statistical significance was determined by Student’s *t*-test (^*^P < 0.05, ^**^P < 0.01). (F) Quantitative analysis of trichome density in plants of different genotypes. (G) Phenotypic comparison of transgenic plants of different genotypes. (a) Whole-plant morphology; (b) close-up views of leaf trichomes; (c) stereomicroscopy images of leaf trichomes; (d) light microscopy images of leaf trichomes; (e) SEM images of leaf trichomes; (f) higher-magnification SEM images of leaf trichomes.

Given that *BcCPR5* expression was significantly lower in W7-2 than in W7-1 (Fig. 4A and Fig. S1), and that VIGS-mediated knockdown of *BcCPR5* in W7-1 substantially reduced trichome density (Fig. 3D), we hypothesized that promoter variation may underlie the reduced trichome density. To test this, we cloned and sequenced the *BcCPR5* promoter region from both parental lines. Sequence alignment revealed a 3365-bp insertion located 473 bp upstream of ATG1 in W7-2, in addition to multiple SNPs and small InDels (Fig. S5). Further annotation indicated that this insertion carries features characteristic of Helitron-N48 and HARB-N19_RSa transposable elements (Fig. S6).

To assess the functional impact of these promoter variants, we compared their transcriptional activity using a dual-luciferase (LUC) reporter assay in *Nicotiana benthamiana*. A 2000-bp fragment upstream of ATG1 from W7-1 (designated *P1*, Fig. 4B) was cloned and fused to the LUC reporter. The corresponding 2000-bp fragment from W7-2, which contains the 3365-bp insertion, was also cloned (designated *P4*, Fig. 4B). In addition, 2000-bp fragments upstream of the downstream ATG2 were cloned from both W7-1 and W7-2 (designated *P3* and *P2*), respectively (Fig*.* 4B). *P1* promoter exhibited strong activity, whereas the activities of *P2*, *P3*, and *P4* were as low as the empty vector control (Fig. 4C and D). These results indicate that variation in the W7-2 promoter, particularly the 3365-bp insertion, drastically reduces promoter activity.

To determine whether this promoter insertion impairs *BcCPR5* function in trichome formation, we generated constructs in which *BcCPR5* was driven by its native promoters from W7-1 (*P1*) and W7-2 (*P2*) and introduced them into the Arabidopsis *cpr5* mutant for complementation assays. Multiple independent transgenic lines were obtained for each construct (Fig. S7). RT-qPCR analysis confirmed that *BcCPR5* was expressed in all transgenic lines; however, expression levels in *P2:: BcCPR5*^W7-2^*(cpr5)* lines were significantly lower than those in *P1:: BcCPR5^W7-1^(cpr5)* (Fig. 4E), consistent with the reduced promoter activity observed in the Dual-LUC assay (Fig. 4D). Phenotypic analysis revealed that *P1::BcCPR5*^W7-1^ lines fully restored trichome density and morphology to wild-type level (Fig. 4F). However, *P2::BcCPR5*^W7-2^*(cpr5)* lines exhibited only partial complementation, that is, the trichome density was higher than that of the *cpr5* mutant but significantly lower than that of *P1*-driven lines (Fig. 4F), and most trichomes were two-branched (Fig. 4G). These findings suggest that the 3365-bp insertion in the W7-2 promoter region is a major determinant of reduced *BcCPR5* expression and, consequently, the low-trichome-density phenotype in W7-2.

### Fine-mapping of a critical region in the *BcCPR5* promoter

**Figure 5:**
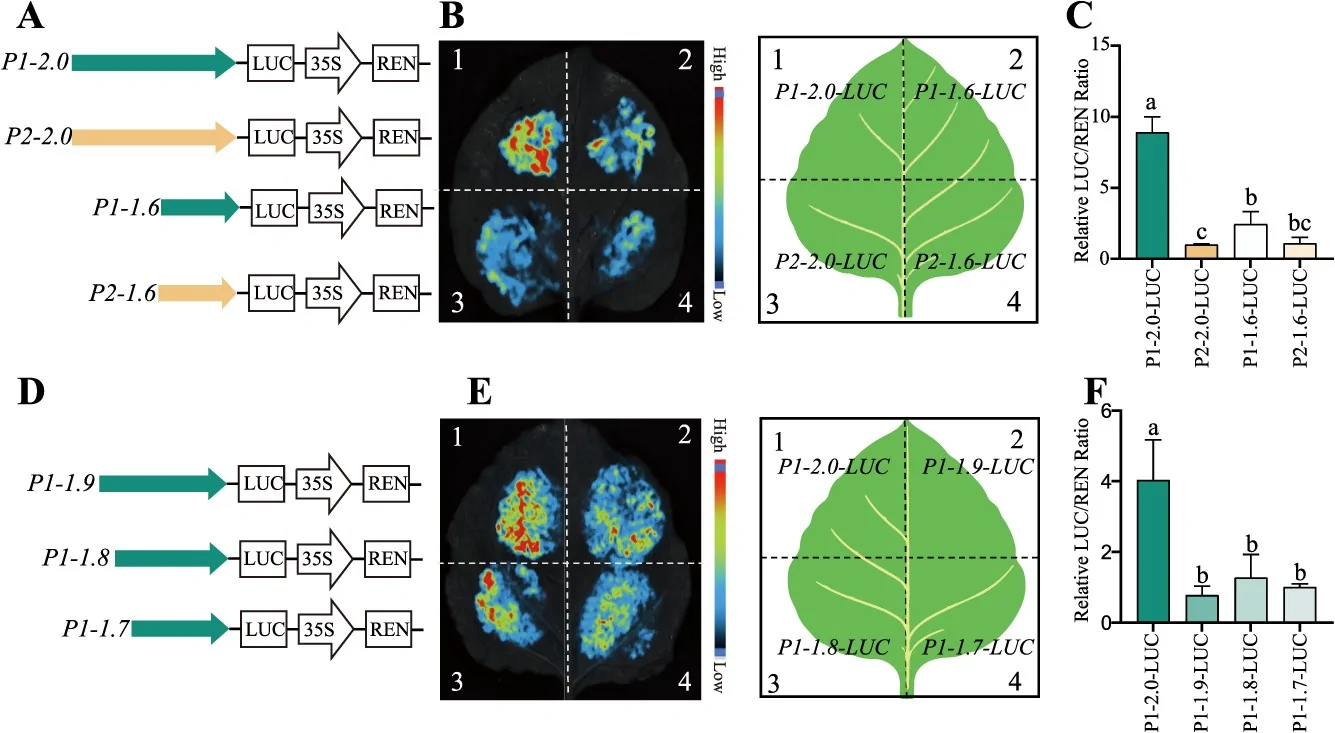
Fine-mapping of critical regions in the *BcCPR5* promoter. (A) Schematic diagram of the truncation strategy for promoter activity analysis of *P1* and *P2*. (B) Luminescence imaging of promoter activity in *N. benthamiana* leaves, comparing promoter activity between *P1* and *P2* for the −2.0 kb fragments, as well as for the −1.6 kb fragments. (C) Quantitative analysis of dual-LUC activity expressed as the LUC/REN ratio. (D) Schematic diagram of the truncation strategy for further promoter activity analysis of the P1 region (−1.7 to −1.9 kb). (E) Luminescence imaging of promoter activity in *N. benthamiana* leaves, comparing promoter activity for truncated fragments of the *P1* region (−1.7 to −2.0 kb). (F) Quantitative analysis of dual-LUC activity expressed as the LUC/REN ratio. Data are presented as mean ± SD (one-way ANOVA, Tukey’s HSD test; different lowercase letters indicate significant differences at *P* < 0.05).

Given that the transcriptional activity of *P3* was significantly lower than that of *P1* (Fig. 4C and D), we proposed that the key regulatory element(s) responsible for *BcCPR5* high expression reside between −1.6 and −2.0 kb region upstream of ATG1. To test this, we first generated a truncated version of *P1* lacking the last 400 bp (*P1-1.6*), with *P2* and *P2* truncation (*P2-1.6*) included as controls (Fig. 5A). LUC reporter assays showed that *P1-2.0* exhibited significantly higher activity than *P1-1.6*, which displayed low activity comparable to *P2-2.0* and *P2-1.6* (Fig. 5B and C). To further pinpoint the active element(s), we conducted progressive deletion across the −2.0 to −1.7 kb region of the *P1* promoter (Fig. 5D). Through this analysis, we localized the strongest enhancer activity of the to a 100-bp region spanning −2.0 to −1.9-kb upstream of ATG1, which displayed higher LUC activity relative to adjacent fragments (Fig. 5E and F). These findings identify a critical enhancer region within the *BcCPR5* promoter that is required for its full transcriptional activity and is likely disrupted by the 3365-bp insertion in W7-2.

### BcWRKY44 acts upstream of *BcCPR5* in trichome formation

**Figure 6:**
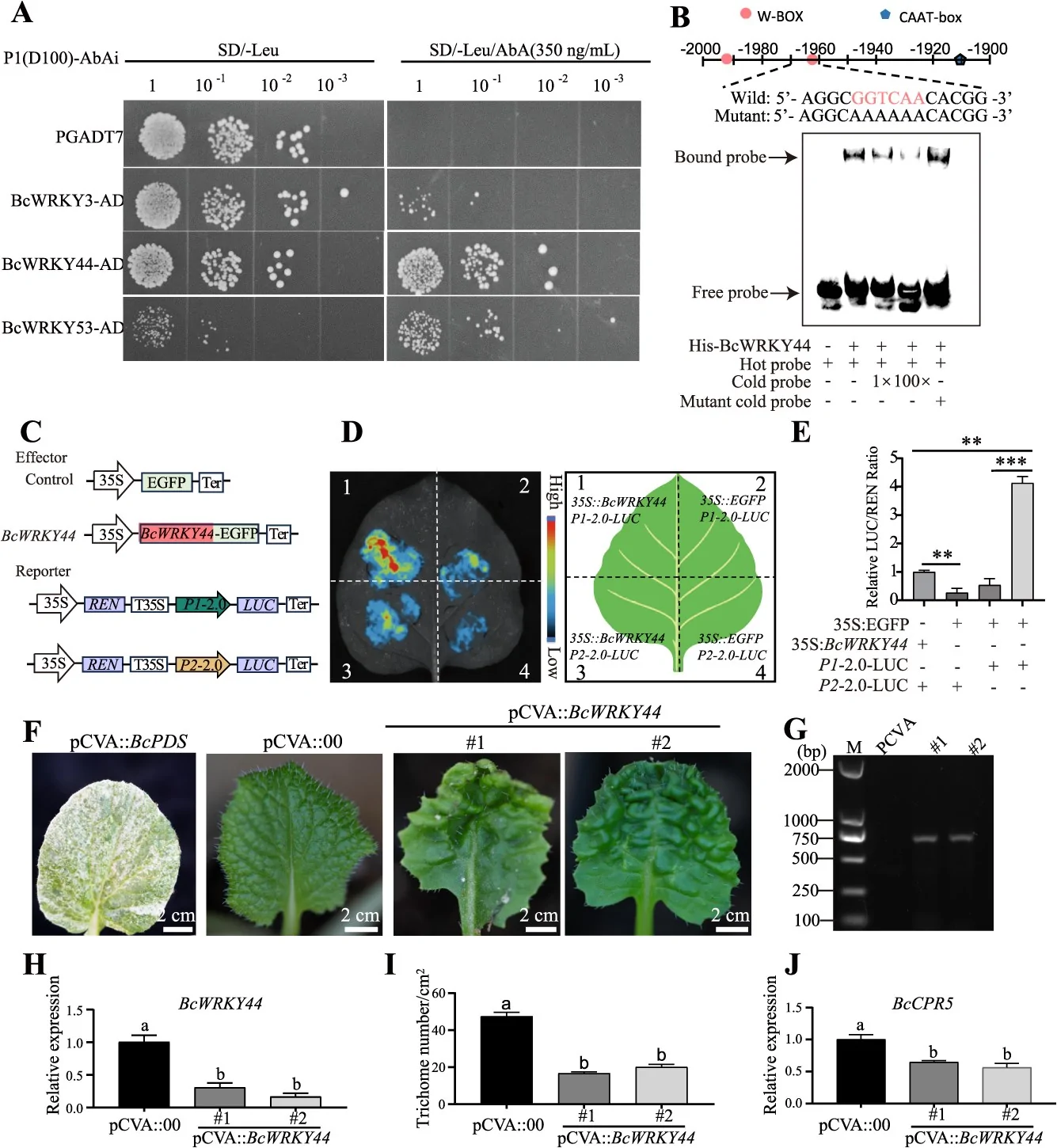
Screening and functional validation of key transcription factor BcWRKY44. (A) Yeast one-hybrid assay using the *P1* fragment (−1.9 to −2.0 kb) as bait. (B) Prediction of cis-acting elements in the −1.9 to −2.0 kb core region of the *P1* promoter, and EMSA demonstrating specific binding of BcWRKY44 to the W-box motif within the *P1* promoter. (C) Schematic diagrams of effector and reporter constructs used for dual-LUC assays. (D) Luminescence comparison of *P1* and *P2* activity with or without *BcWRKY44* coexpression. (E) Dual-LUC activity assays. Data are presented as mean ± SD (Student’s *t*-test, ^**^P < 0.01, ^***^P < 0.001). (F) Phenotypes of *BcWRKY44*-silenced plants generated by VIGS. (G) PCR-based genotyping confirmation of silencing efficiency in VIGS lines. (H) Relative transcript levels of *BcWRKY44* in VIGS lines determined by RT-qPCR. (I) Comparison of trichome density between *BcWRKY44*-VIGS lines and pCVA::00 controls. (J) Relative transcript levels of *BcCPR5* in *BcWRKY44*-VIGS lines determined by RT-qPCR. Data are presented as mean ± SD (one-way ANOVA with Tukey’s HSD test; different lowercase letters indicate significant differences at *P* < 0.05).

To identify upstream regulators of *BcCPR5*, we performed a yeast one-hybrid screening using the 100-bp enhancer region as bait. This screen identified three WRKY transcription factors, namely, BcWRKY53, BcWRKY44, and BcWRKY3, among which BcWRKY44 showed the strongest binding activity to the bait fragment (Fig. 6A). Functional analysis of the corresponding Arabidopsis mutants revealed that only the *atwrky44* mutant exhibited a significant reduction in trichome density (Figs S8A, B and S9A, B). Sequence alignment showed that BcWRKY44 shares ~80% amino acid identity with AtWRKY44 (Fig. S8C), indicating functional conservation in trichome regulation. Therefore, we focused subsequent analyses on BcWRKY44.

Dual-LUC reporter assays demonstrated that coexpression of *BcWRKY44* significantly enhanced the activity of the *P1* promoter but had no effect on *P2* (Fig. 6C–E). To pinpoint the responsive cis-element(s), we examined the −2.0 to −1.9 kb region of *P1* and identified two canonical W-box motifs (Fig. 6B). Electrophoretic mobility shift assay (EMSA) confirmed that the recombinant His-BcWRKY44 protein directly bound to a biotin-labeled probe containing this W-box. The binding was competitively inhibited by an excess of unlabeled wild-type probe but not by a mutated version, demonstrating sequence-specific binding (Fig. 6B).

To functionally validate the role of *BcWRKY44* in trichome formation in wucai, we performed VIGS in the high-trichome-density line W7-1 (Fig. 6F). A 472-bp fragment from the 3′ end of *BcWRKY44* was cloned into the pCVA vector. Four weeks after infiltration, viral vector accumulation was confirmed in pCVA::*BcWRKY44*-infiltrated plants (Fig. 6G). RT-qPCR analysis revealed that *BcWRKY44* transcript levels were significantly reduced in these lines compared to empty vector controls (Fig. 6H), confirming effective silencing. Concomitantly, trichome density was markedly reduced on leaves of *BcWRKY44*-silenced plants, whereas empty vector controls maintained high trichome density (Fig. 6I). Notably, *BcCPR5* transcript levels were also significantly downregulated in the *BcWRKY44*-silenced lines (Fig. 6J). Taken together, these findings demonstrate that BcWRKY44 directly activates *BcCPR5* transcription by binding to a W-box element in its promoter and plays an essential, evolutionarily conserved role in regulating trichome density in wucai.

### Population genetic analysis of *BcCPR5* structural variants in *Brassica* species

**Figure 7:**
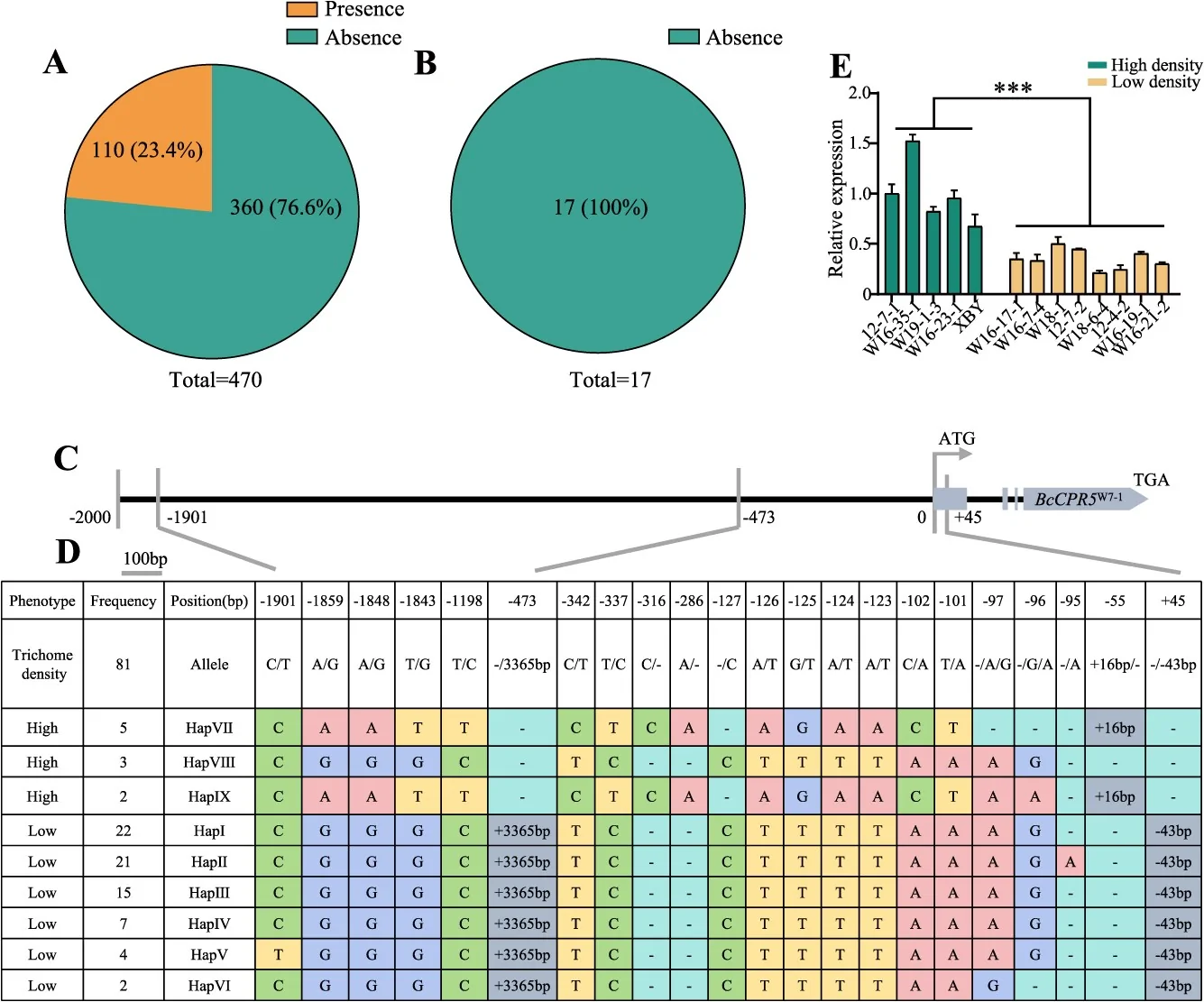
Allelic distribution and expression analysis of *BcCPR5* in wucai. (A) Distribution of individuals with and without promoter insertion variation in low-density-trichome wucai and glabrous pakchoi populations. (B) Proportion of individuals without promoter insertion variation in high-trichome-density wucai population. (C) Schematic diagram depicting the distribution of SNPs and InDels in the *BcCPR5* promoter region. (D) Haplotype analysis of the promoter region in 10 wucai cultivars with high trichome density and 71 wucai cultivars with low trichome density. (E) Relative *BcCPR5* expression levels in leaves of five high-trichome-density and eight low-trichome-density wucai cultivars, determined by RT-qPCR (*n* = 3).

To assess the distribution of the 3365-bp promoter insertion and its association with trichome density, we genotyped a diverse panel consisting of 408 modern wucai inbred lines and 79 pakchoi accessions (Table S6). The insertion was detected exclusively in low-trichome-density materials: it was present in 7.5% (10/134) of low-trichome-density heterozygous wucai lines and 27.6% (71/257) of low-density homozygous lines. Among pakchoi lines, the insertion frequency researched 36.7% (29/79). Overall, the average frequency of this promoter variant across low-trichome-density and glabrous wucai and pakchoi populations was ~23.4% (Fig. 7A). In contrast, none of the high-trichome-density lines, including 10 homozygous and 7 hybrid lines, harbored the insertion in the *BcCPR5* promoter (Fig. 7B).

To further confirm that the 3365-bp insertion, rather than other linked polymorphisms, is responsible for reduced *BcCPR5* expression and low trichome density in natural populations, we performed detailed promoter sequence analysis in 81 wucai cultivars, comprising 10 high-density and 71 low-density accessions. Nine major haplotypes (HAP1–HAP9) were identified in the *BcCPR5* promoter region (Fig. 7C and D). Comparison of the haplotypes between high- and low-density groups revealed that the absence of the 3365-bp insertion was the most consistent feature distinguishing high-density accessions. Moreover, *BcCPR5* transcript levels were significantly higher in high-density accessions than in low-density cultivars (Fig. 7E). These findings provide strong evidence that the 3365-bp promoter insertion contributes to reduced *BcCPR5* expression and, consequently, decreased trichome density in wucai.

## Discussion

Trichomes serve as essential physical defense structures in plants and are widely deployed during plant growth and development [34]. Despite the versatility, our multiyear field observation and another report [24] reveal that low trichome density predominates in modern cultivated wucai and other non-heading Chinese cabbage populations (Table S6). This intriguing phenomenon prompted us to explore the molecular mechanisms underlying trichome loss in wucai.

**Figure 8:**
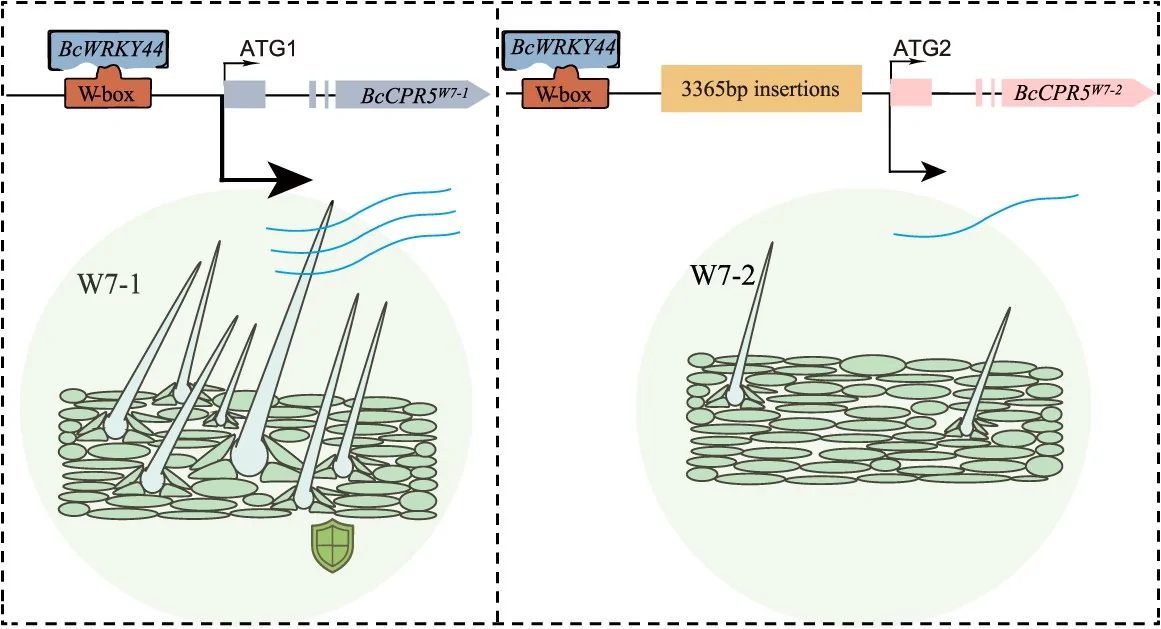
Proposed model for transposon insertion-mediated regulation of *BcCPR5* expression *via* BcWRKY44.

Based on our findings, we propose a model illustrating the regulatory mechanism governing trichome density variations between the wucai inbred lines W7-1 and W7-2 (Fig. 8). In W7-1, the transcription factor BcWRKY44 binds specifically to a W-box cis-element in the *BcCPR5* promoter, activating its expression and leading to high trichome density, which likely confers biotic and/or abiotic stress resistance. In W7-2, a 3365-bp transposon insertion in the promoter disrupts BcWRKY44 binding and/or transcriptional ability, resulting in reduced *BcCPR5* transcription and a consequently lower trichome density (Fig. 8).

RT-qPCR analysis showed that *BcCPR5* expression in W7-2 was only ~40% lower than in W7-1 (Fig. 4A), and this modest reduction was tightly associated with the promoter insertion and low-trichome-density phenotype. A similar trend was reported in cotton, where reducing *GheCPR5.1* expression to 50% of wild-type level significantly decreases trichome density [35]. The apparent discrepancy between the relatively modest expression reduction and the pronounced phenotypic effect may be explained by cell-type-specific expression. *BcCRP5* may be preferentially expressed in epidermal cells, where trichome development occurs, or even within trichome cells themselves, rather than the whole leaf tissue, like its downstream target genes *SIM* and *SMR1*, and other well-characterized trichome regulators [36–39]. This interpretation is supported by dual-LUC assays, which showed that the 3365-bp transposon insertion reduces *BcCRP5* promoter activity by ~80% (Fig. 4D). Thus, the reduction in transcript level may be diluted in whole-leaf samples used for RT-qPCR, leading to an underestimation of the actual suppression in trichome cells. Further studies employing *in situ* hybridization or GUS reporter assays would help clarify this issue.

Both in the native W7-2 lines (Fig. 1B) and in complementation assays using native promoters (Fig. 4A), trichome density correlated positively with *BcCPR5* expression level. However, in 35S-driven complementation assays, all transgenic lines, despite variable expression levels, fully rescued the *cpr5* mutant phenotype without further enhancement (Fig. S4), consistent with previous observations in Arabidopsis [40]. We propose a threshold-dependent model for CPR5 function in trichome formation. In W7-2, the transposon insertion reduces *BcCPR5* expression below the critical threshold required for normal trichome formation.

In Arabidopsis, *CPR5* influences trichome development by modulating cell cycle progression [30]. The *cpr5* mutant exhibits pleiotropic cellular defects, including smaller cell size, decreased endoreduplication levels [41], and reduced trichome branching [36]. In wucai, trichomes are unbranched (Fig. 1D and E), which precludes assessment of branching function. Nevertheless, *BcCPR5* fully restored trichome density and morphology in the Arabidopsis *cpr5* mutant (Fig. S4), indicating functional conservation of CPR5 in trichome regulation between Arabidopsis and Wucai. Notably, trichome density in wucai is substantially lower than in Arabidopsis (Figs 1B and 4F), which may explain why loss of *BcCPR5* results in a near-glabrous phenotype, a more striking effect than in the Arabidopsis *cpr5* mutant.

Although CPR5 function has been characterized in Arabidopsis for nearly three decades [31], the upstream regulatory mechanism controlling *CPR5* expression during trichome development has remained largely unclear. In this study, we identified BcWRKY44 as a direct upstream activator of *BcCPR5*, binding specifically to a W-box cis-element located between −1960 and −1964 bp in the P1 promoter (Fig. 6B). The observation that the Arabidopsis *wrky44* mutant displays trichome defects (Fig. S8A) suggests that the WRKY44-CPR5 regulatory module is likely conserved across plants. Moreover, we noticed that knockdown of either *BcCPR5* or *BcWRKY44 via* VIGS also led to wrinkled leaves in wucai (Figs 3A and 6F), but not in the corresponding Arabidopsis mutants (Fig. 4G and Fig. S8A). A similar inconsistent phenotype has been observed upon disruption of certain cell cycle-associated genes [42, 43]. For instance, knockdown of *Block of proliferation 1* in *Gossypium hirsutum* results in wrinkled leaves, but not in Arabidopsis [37]. This finding indicates that the BcWRKY44-BcCPR5 module may function in cell cycle regulation not only in trichome cells but also in other cell types, such as epidermal cells, in wucai.

Analysis of 487 non-heading Chinese Cabbage accessions revealed that only 3.49% of modern cultivated varieties retain high-trichome-density (Table S6). This pattern reflects breeding preference in leafy vegetables, where low trichome density facilitates manual harvesting and aligns with consumer demands. The 3365-bp insertion in the *BcCPR5* promoter is present in 27.63% of inbred wucai lines and 36.7% in fully glabrous pakchoi, suggesting it represents a selective variant rather than a random mutation. Moreover, the findings indicate that, in addition to *BcCPR5*, other genes may have contributed to the domestication process of trichome density in non-heading Chinese Cabbage. Although the 43-bp deletion does not affect *BcCPR5* function in trichome density (Fig. S4), it is completely linked with the 3365-bp insertion (Fig. 7D), making it a potentially useful molecular marker for trichome selection.

Plant trichomes function as natural protective barriers against biotic and abiotic stresses. Given increasing public health concerns over pesticide overuse and the rising frequency of extreme weather events, breeding non-heading Chinese cabbage with appropriate trichome density could represent a sustainable cultivation strategy. Beyond their defensive role, trichomes may also offer nutritional value. Studies in tea have shown that trichomes, though unicellular, accumulate water-soluble metabolites—including free catechins, caffeine, and amino acids—and serve as key quality indicators influencing both aroma and infusion color [44, 45]. Future breeding efforts should employ widely-targeted metabolomics to explore the nutritional potential of high-density trichomes in wucai, while also leveraging genomics-based approaches to uncover additional structural variations linked to agronomic traits [46, 47]. Such strategies will facilitate the full utilization of germplasm resources, enabling the development of new varieties with enhanced nutritional profiles and market appeal.

This study systematically elucidates the genetic and molecular mechanisms underlying trichome formation in wucai. SEM revealed that wucai trichomes are unicellular, unbranched, and nonglandular prickles. Genetic analysis indicated that the trait is controlled by a single dominant nuclear gene, as evidenced by a 3:1 segregation ratio in the F_2_ population. Map-based cloning identified a 114.06-kb critical region on chromosome A06, and *BcCPR5* was confirmed as the key gene regulating trichome density. Functional complementation assays showed that all *BcCPR5* coding-sequence variants fully restored trichome density in the Arabidopsis *cpr5* mutant, whereas a 3365-bp insertion in the W7-2 promoter significantly suppressed transcriptional activity and reduced trichome density. Mechanistically, BcWRKY44 specifically binds to a W-box cis-element at the −1960 to −1964 region of the P1 promoter to activate its expression, and this binding site is essential for maintaining high transcriptional activity. Population analysis further confirmed the absence of the 3365-bp insertion in all high-trichome-density accessions, supporting its potential role in breeding selection.

## Materials and methods

### Plant growth and phenotypic analysis

Two wucai inbred lines, W7-1 and W7-2, were selected as parental lines to generate segregating populations. Both lines had undergone >10 generations of self-pollination to ensure genetic homozygosity. The F_1_ population was obtained by crossing W7-1 (♀) with W7-2 (♂), and subsequent self-pollination of the F_1_ plants produced the F_2_ mapping population. Plants were cultivated under controlled greenhouse conditions at Anhui Agricultural University (Hefei, China) with temperatures maintained at 25°C and a 16/8 h light/dark photoperiod.

### Quantification of trichome density

For plants with ambiguous trichome patterns, trichome density (trichomes/cm^2^) was quantified microscopically using a CNOPTEC SZ650 stereomicroscope (0.7–4.5× magnification, Chongqing Optec Instrument Co., Ltd, China). Starting at the two-leaf-one-heart stage (14 days after sowing), three mature leaves per plant were examined, with trichome counts performed in three 1-cm^2^ areas along the leaf margin [48].

### Scanning electron microscopy analysis

Fresh leaf segments (5 × 5 mm) from the plant were excised perpendicular to the major veins using a sterile surgical blade and immediately fixed in 4% (v/v) glutaraldehyde at room temperature for ≥2 h. After fixation, the samples were rinsed four times with 0.1 M phosphate buffer (pH 6.8) at 10-min intervals for three times. Dehydration was carried out through a graded ethanol series (30%, 50%, 70%, 80%, 90%, and two consecutive 100% ethanol treatments), with each step lasting 10 min. Critical point drying was performed using liquid CO_2_ (Emitech K850, England). The dried samples were subsequently sputter-coated with gold (Hitachi E1010 Ion Sputter, Japan) and imaged under high vacuum at an accelerating voltage of 3 kV using a field emission scanning electron microscope (SEM) (Hitachi S-4800, Japan).

### Bulked segregant analysis sequencing analysis

Genomic DNA was extracted from fresh leaves using the CTAB method [49], and DNA quality was evaluated by spectrophotometric measurement and 1% agarose gel electrophoresis. Two extreme bulks were constructed by mixing equal amounts of DNA from 30 F_2_ individuals with high trichome density and 30 F_2_ individuals with low trichome density, respectively. Genomic DNA from five plants of each parental line was also collected.

For library preparation, genomic DNA was fragmented to an average size of 350–500 bp using a Covaris S220 Focused-ultrasonicator, followed by adapter ligation using the TruSeq DNA LT Sample Prep Kit (Illumina, San Diego, CA, USA). After PCR amplification and purification, libraries were subjected to 150 bp paired-end sequencing on an Illumina HiSeq X Ten platform.

Raw sequencing reads were filtered using fastp (v0.19.5). Clean reads were aligned to the *B. rapa* reference genome (https://ftp.ncbi.nlm.nih.gov/genomes/all/GCF/000/309/985/GCF_000309985.2_CAAS_Brap_v3.01/) using BWA (v0.7.12) [50, 51]. SAMtools (v1.4) was used for sorting and indexing [52], and PCR duplicates were removed with Picard Tools (v4.1.0.0). Variant calling (SNPs and InDels) was performed using GATK (v4.1.0.0) [53], and variants were annotated with SnpEff [54].

Two parameters, SNP-index and Δ(SNP-index), were calculated to identify candidate regions for wucai trichome gene [55]. A sliding window analysis (1 Mb window size, 10 kb increment) was applied to plot genome-wide distributions. Confidence intervals for Δ(SNP-index) were generated under the null hypothesis of no QTL, and positions exceeding the 95% confidence threshold were considered significantly associated with the trait.

### Development of molecular markers and genotyping analysis

To further refine the candidate region identified through BSA-seq analysis, InDel markers targeting genomic intervals containing insertions or deletions of at least 20 bp were developed. Primers were designed using Oligo7 software (primer sequences provided in Table S7) and used for genotyping the F_2_ mapping population.

PCR amplification was carried out under standardized conditions, and the products were separated on 8% denaturing polyacrylamide gels followed by silver staining. Markers displaying clear codominant polymorphisms and distinct banding patterns in both parents and F_1_ hybrids were selected for subsequent analysis. These validated InDel markers were then employed to detect recombination events and progressively refine the target genomic interval through linkage analysis in the F_2_ population.

### Virus-induced gene silencing in wucai

To investigate the function of *BcCPR5* and BcWRKY44 in wucai, VIGS assays were performed using the CaLCuV-based vectors pCVA and pCVB [33].

For *BcCPR5* silencing, a 350-bp fragment from the 3′ untranslated region of the *BcCPR5* gene was PCR amplified from W7-1 cDNA using gene-specific primers (Table S4). The resulting PCR product was digested with *Kpn*I and *Xba*I and cloned into the corresponding sites of pCVA to generate pCVA-*BcCPR5*.

For *BcWRKY44* silencing, a 472-bp fragment from the 3′ end of the *BcWRKY44* CDS was PCR amplified from W7-1 cDNA. The fragment was digested with *Kpn*I and *Xba*I and ligated into the same sites of pCVA, yielding pCVA-*BcWRKY44*. All constructs were confirmed by DNA sequencing.

The recombinant pCVA constructs were individually introduced into *Agrobacterium tumefaciens* strain GV3101 by electroporation. For VIGS assays, *Agrobacterium* cultures harboring pCVA-derived constructs and pCVB were mixed at a 1:1 ratio (OD_600_ = 1.0 each) and infiltrated into the fully expanded cotyledons of wucai seedlings using a needleless syringe. Plants infiltrated with empty pCVA and pCVB served as negative controls, while those infiltrated with pCVA-*BcPDS* (phytoene desaturase) served as positive controls for silencing efficiency. Four weeks after infiltration, newly emerged leaves were collected for phenotypic observation and molecular analyses.

### RNA extraction, reverse transcription, and RT-qPCR

Total RNA was isolated from each sample using the TaKaRa MiniBEST Plant RNA Extraction Kit (Takara, Japan) according to the manufacturer’s protocol. Subsequently, 1 μg of total RNA from each sample was reverse-transcribed into cDNA using the PrimeScript™ FAST RT Reagent Kit with gDNA Eraser (Takara, Japan), following the manufacturer’s instructions.

RT-qPCR was conducted on the CFX96 Real-Time PCR Detection System (Bio-Rad, Hercules, CA, USA) using the SupRealQ Purple Universal SYBR qPCR Master Mix (U+) (Vazyme, Nanjing, China). Each reaction was performed in technical triplicate alongside appropriate negative controls. Thermal cycling was carried out according to the manufacturer’s instructions for the SYBR Green Master Mix. Relative gene expression levels were quantified using the 2^−ΔΔCt^ method, with the Chinese cabbage *ACTIN* gene (*BcActin*) serving as the internal reference standard. All primer sequences employed in the RT-qPCR analysis are listed in Table S7.

### Dual-luciferase reporter assay

To analyze the promoter activity of *BcCPR5*, the 1.6-kb and 2.0-kb promoter regions of *BcCPR5*^W7-1^ and *BcCPR5*^W7-2^ were amplified from both parental lines and cloned into the pGreenII 0800-LUC vector to generate the reporter constructs *P1-2.0-LUC*, *P2-2.0-LUC*, *P3-2.0-LUC*, *P1-1.6-LUC*, and *P2-1.6-LUC*. To further identify key regulatory sequences responsible for enhancing promoter *P1* activity, fragments of *P1-1.7*, *P1-1.8*, and *P1-1.9* were cloned into the same vector. The 2.0-kb promoter fragments of *P1* and *P2* were cloned into the pGreenII0800-LUC vector as reporters.

To investigate whether BcWRKY44 binds to the *BcCPR5* promoter, dual-LUC reporter assays were performed. The full-length CDS of *BcWRKY44* was cloned into the pCAMBIA2300 vector under the control of the CaMV 35S promoter to generate the effector construct. For reporter constructs, the 2.0-kb promoter fragments of *P1* and *P2* were amplified and cloned into the pGreenII 0800-LUC vector.

For transient expression assays, all constructs were introduced into *A. tumefaciens* strain GV3101 along with the helper plasmid pSoup. *N. benthamiana* leaves were infiltrated with these *Agrobacterium* cultures carrying the reporter constructs. Leaf samples were harvested 48 h after infiltration for analysis. LUC activity was quantified using the Dual Luciferase Reporter Assay Kit (Vazyme, Nanjing, China), and the measurements were normalized to Renilla (REN)-LUC as an internal control. Each construct was evaluated in three biological replicates. All primer sequences used for vector construction are provided in Table S7.

### Yeast one-hybrid library screening and transcription factor identification

A fragment spanning the −1.9 to −2.0 kb region of the *BcCPR5* promoter was cloned into the pAbAi vector and transformed into the Y1HGold yeast strain. Successful genomic integration was confirmed using the FTL MIX Kit (Mei5 Biotechnology, Beijing, China). The minimum inhibitory concentration of aureobasidin A (AbA) was determined to be 350 ng/ml. The bait strain was cotransformed with a pGADT7-based cDNA library and plated on SD/-Leu medium supplemented with 350 ng/ml AbA. Positive clones were identified, sequenced, and compared against the *B. rapa* v3.01 (chifu) genome database. Candidate transcription factors were further verified by cloning their full-length CDS into pGADT7 and cotransforming with the bait construct, using the empty pGADT7 vector as a negative control. All primers used in these experiments are listed in Table S7.

### Electrophoretic mobility shift assay

The full-length CDS of *WRKY44* was cloned into the pCold vector and transformed into Rosetta (DE3) competent cells. Protein expression was induced with 0.5 mM IPTG at 16°C for 12 h. The resulting 6× His-tagged recombinant protein was purified using BeyoGold™ His-tag Purification Resin (Beyotime, Shanghai, China), and protein purity was verified by 6% SDS-PAGE and Coomassie Brilliant Blue staining. The subsequent protein-probe binding assay was performed as previously described [56], using Chemiluminescent EMSA Kit (Beyotime, Shanghai, China). The biotin-labeled oligonucleotide probes used in these assays are listed in Table S7.

## Supplementary Material

Web_Material_uhag184

## Data Availability

All relevant data can be found within the article and its supporting materials.
