# Supplementary material for: A transposon insertion in the promoter of BcCPR5 impairs trichome formation in wucai (Brassica campestris L.)
Source: Hortic Res. 2026 May 8;13(10):uhag184. doi: 10.1093/hr/uhag184 (PMC13610597; doi:10.1093/hr/uhag184)
Supplement: Supplementary file 1 [file web_material_uhag184.zip › FigureS1.pdf]

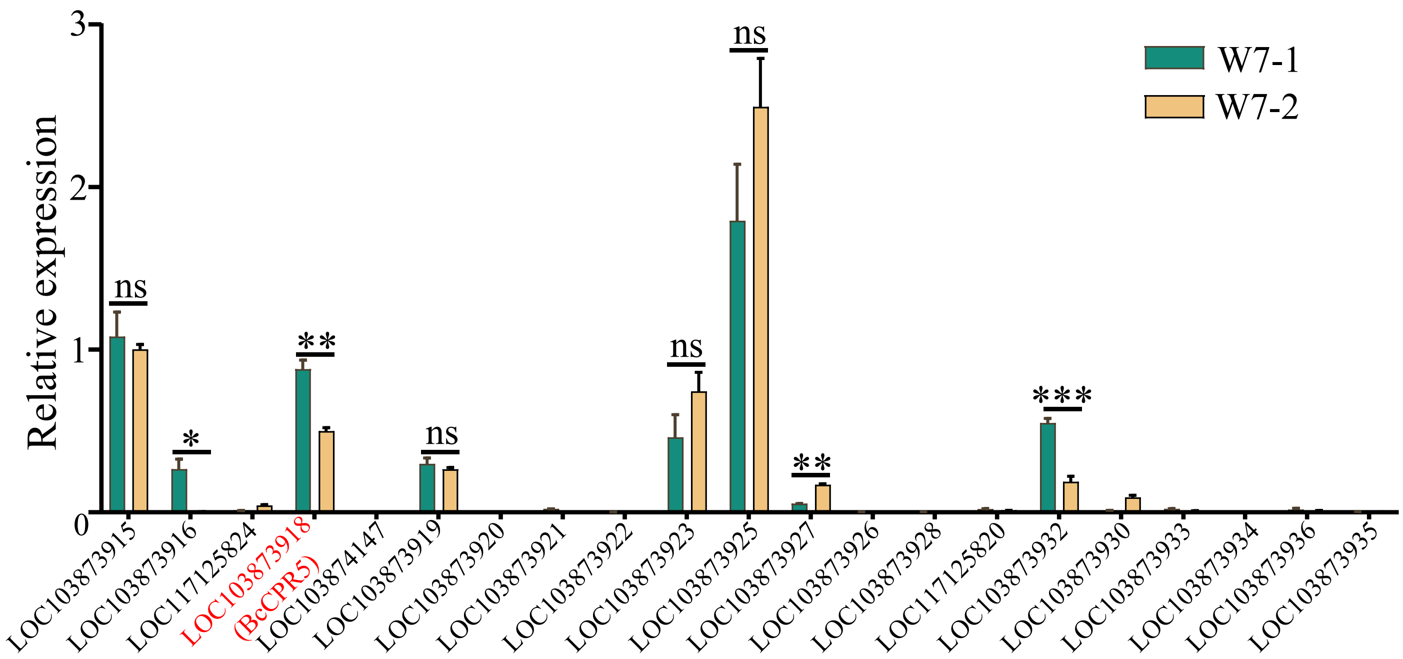

Figure S1. Relative expression levels of the 21 predicted genes in the candidate region determined by quantitative real-time PCR (RT-qPCR). Data are presented as mean ± SD (Student's t test, *$P<0.05$, **$P<0.01$, *** $p < 0.001$; ns, non-significant)
