# Supplementary material for: A transposon insertion in the promoter of BcCPR5 impairs trichome formation in wucai (Brassica campestris L.)
Source: Hortic Res. 2026 May 8;13(10):uhag184. doi: 10.1093/hr/uhag184 (PMC13610597; doi:10.1093/hr/uhag184)
Supplement: Supplementary file 1 [file web_material_uhag184.zip › FigureS2.pdf]

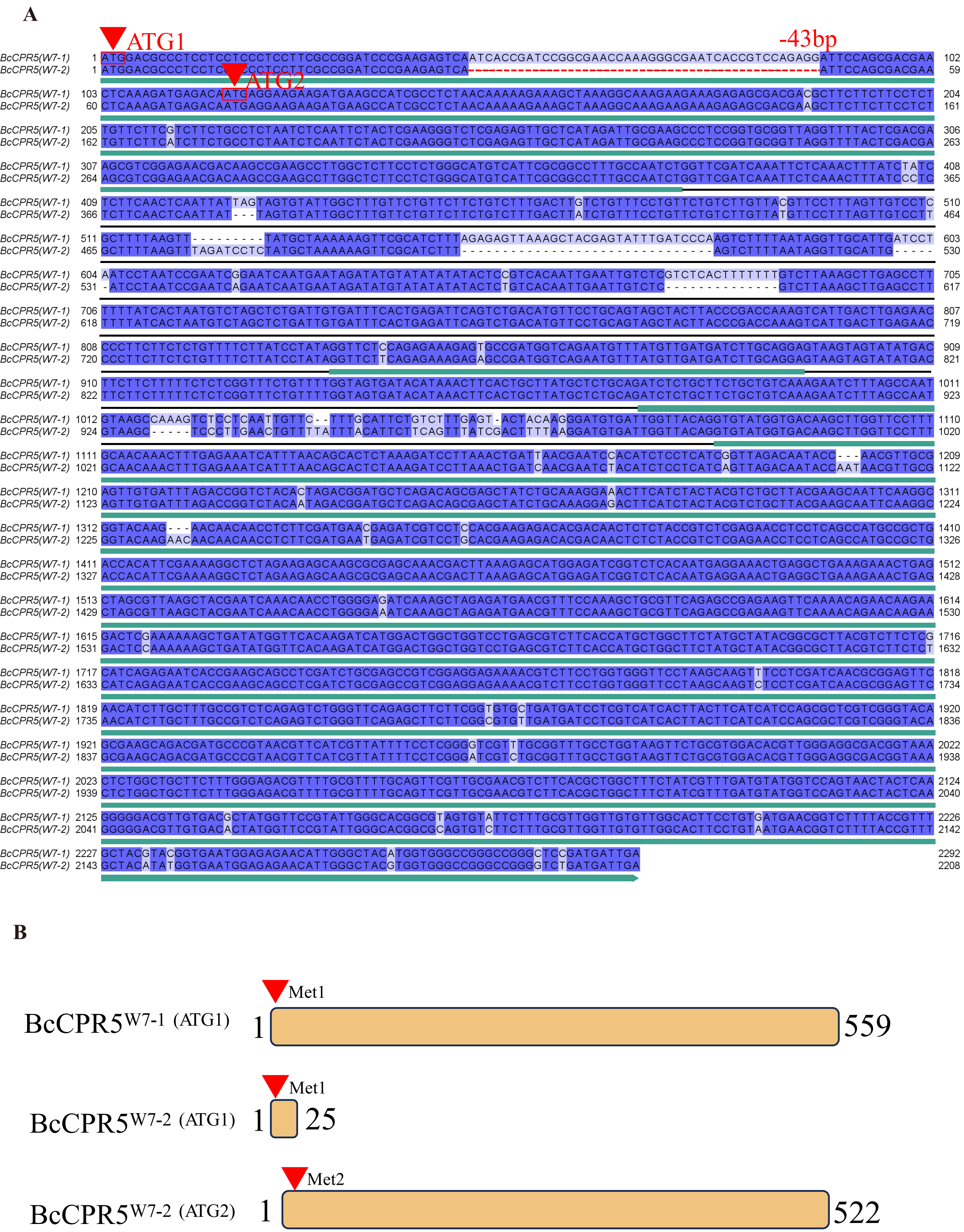
A
ATG1
ATG2
-43bp
BcCPR5(W7-1)
BcCPR5(W7-2)
B
Met1
BcCPR5W7-1 (ATG1)
1
559
Met1
BcCPR5W7-2 (ATG1)
1
25
Met2
BcCPR5W7-2 (ATG2)
1
522

Figure S2. Comparative analysis of *BcCPR5* genomic DNA and protein sequences. (A) Comparative analysis of the genomic DNA sequence of *BcCPR5*. Solid green boxes indicate exons, solid black boxes indicate introns, and the red dashed line marks the 43-bp deletion region resulting from natural variation. Two start codons are indicated by red boxes and labels: ATG1 denotes the first start codon annotated in the reference genome, while ATG2 represents a potential alternative start codon. (B) Schematic diagram of the amino acid sequences of different BcCPR5 proteins; $BcCPR5^{W7\text{-}1\ (ATG1)}$: the BcCPR5 protein translated from ATG1 in W7-1, consisting of 559 amino acids; $BcCPR5^{W7\text{-}2\ (ATG1)}$: the BcCPR5 protein translation initiated at ATG1 in W7-2, which undergoes premature translation termination due to the 43-bp natural deletion, resulting in only 25 amino acids; $BcCPR5^{W7\text{-}2\ (ATG2)}$: the BcCPR5 protein translated from ATG2, consisting of 522 amino acids.
