# Supplementary material for: A transposon insertion in the promoter of BcCPR5 impairs trichome formation in wucai (Brassica campestris L.)
Source: Hortic Res. 2026 May 8;13(10):uhag184. doi: 10.1093/hr/uhag184 (PMC13610597; doi:10.1093/hr/uhag184)
Supplement: Supplementary file 1 [file web_material_uhag184.zip › FigureS3.pdf]

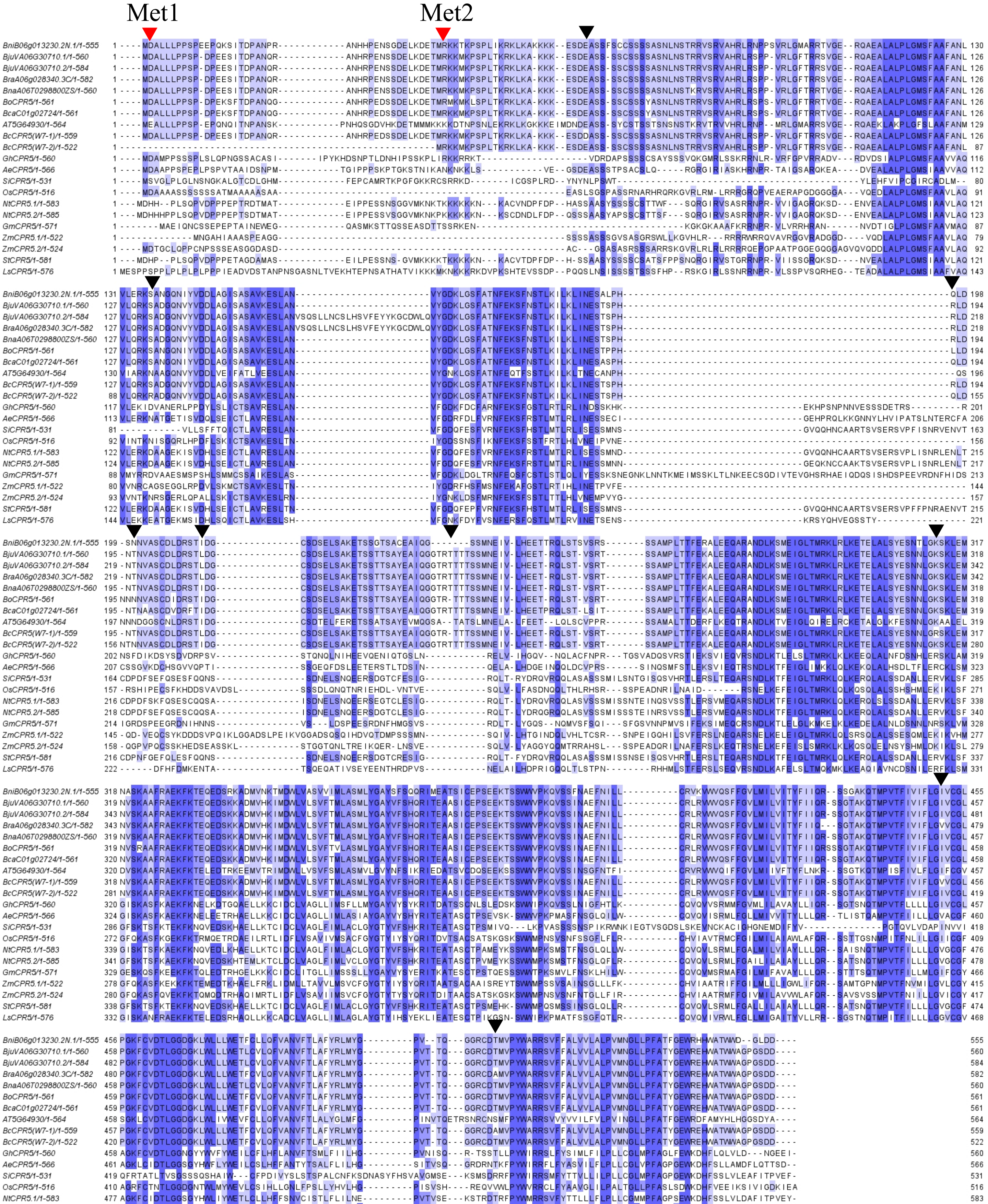

Figure S3. Comparative analysis of BcCPR5 protein amino acid sequences across different species. Red downward arrows indicate the positions of Met1 and Met2 generated from two distinct start codons; black downward arrows mark the positions of amino acid changes resulting from missense mutations in W7-1 and W7-2.
