# Supplementary material for: A transposon insertion in the promoter of BcCPR5 impairs trichome formation in wucai (Brassica campestris L.)
Source: Hortic Res. 2026 May 8;13(10):uhag184. doi: 10.1093/hr/uhag184 (PMC13610597; doi:10.1093/hr/uhag184)
Supplement: Supplementary file 1 [file web_material_uhag184.zip › FigureS4.pdf]

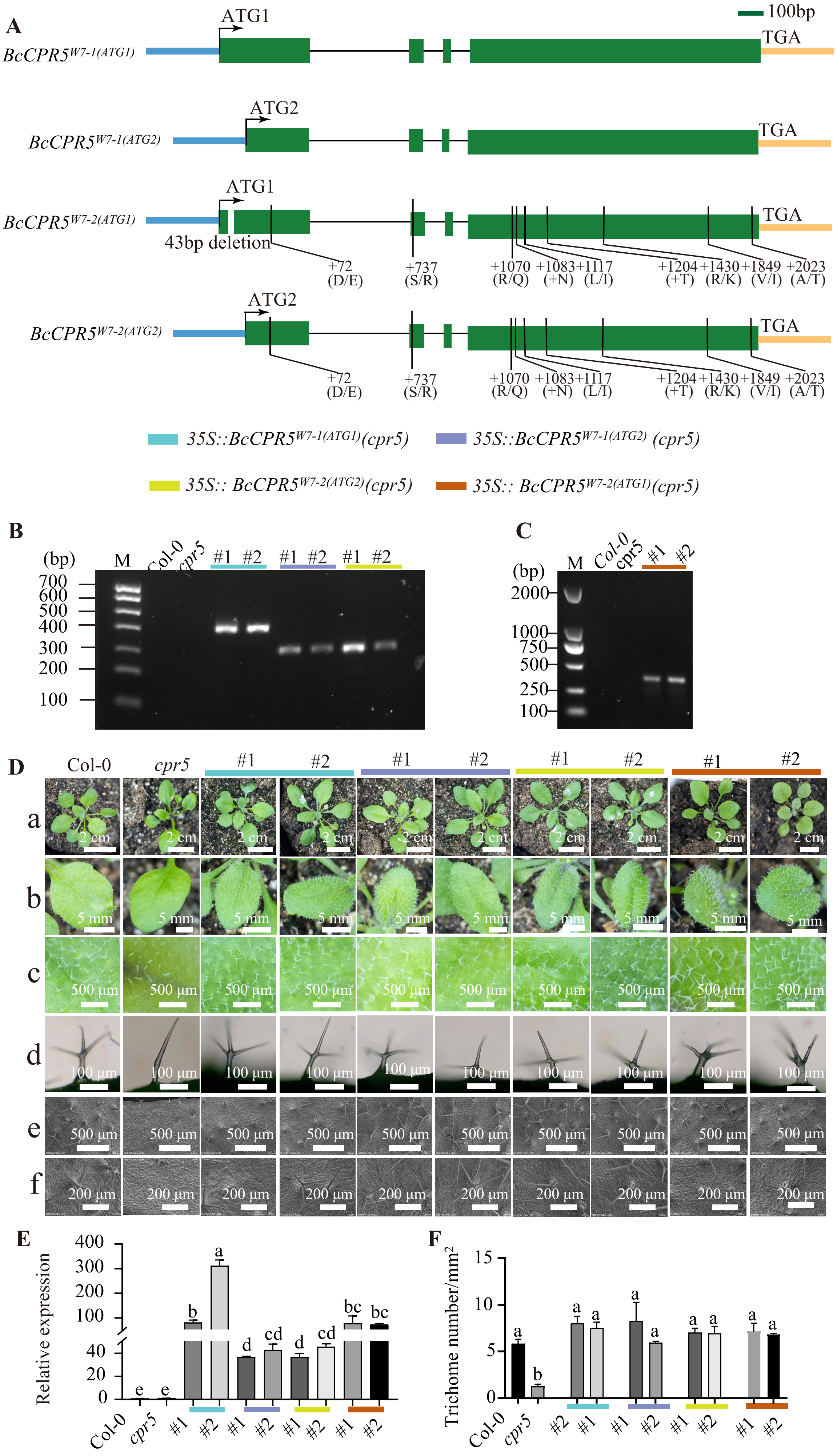
A
B
C
D
E
F
100bp
ATG1
ATG2
TGA
43bp deletion
+72 (D/E)
+737 (S/R)
+1070 (R/Q)
+1083 (+N)
+1117 (L/I)
+1204 (+T)
+1430 (R/K)
+1849 (V/I)
+2023 (A/T)
35S::BcCPR5W7-1(ATG1)(cpr5)
35S::BcCPR5W7-1(ATG2) (cpr5)
35S:: BcCPR5W7-2(ATG2)(cpr5)
35S:: BcCPR5W7-2(ATG1)(cpr5)
(bp)
M
Col-0
cpr5
#1
#2
Relative expression
Trichome number/mm2
2 cm
5 mm
500 μm
100 μm
200 μm

Figure S4. Functional validation of the *BcCPR5* (A) Schematic diagram of the four *BcCPR5* gene structures resulting from the presence of ATG1 and ATG2 in W7-1 and W7-2. Green boxes represent exons, and thin black lines represent introns. (B) PCR-based genotyping confirmation of transgenic plants carrying *35S::BcCPR5*$^{W7\text{-}1(ATG1)}$, *35S::BcCPR5*$^{W7\text{-}1(ATG2)}$, and *35S::BcCPR5*$^{W7\text{-}2(ATG2)}$. (C) Relative expression levels of *CPR5* in transgenic lines carrying *35S::BcCPR5*$^{W7\text{-}1(ATG1)}$, *35S::BcCPR5*$^{W7\text{-}1(ATG2)}$, and *35S::BcCPR5*$^{W7\text{-}2(ATG2)}$. (D) Quantitative analysis of trichome density in transgenic lines carrying *35S::BcCPR5*$^{W7\text{-}1(ATG1)}$, *35S::BcCPR5*$^{W7\text{-}1(ATG2)}$, and *35S::BcCPR5*$^{W7\text{-}2(ATG2)}$. (E) PCR-based genotyping confirmation of transgenic plants carrying *35S::BcCPR5*$^{W7\text{-}2(ATG1)}$ in the *cpr5* mutant background. (F) Relative expression levels of BcCPR5 in transgenic lines carrying *35S::BcCPR5*$^{W7\text{-}2(ATG1)}$. (G) Quantitative analysis of trichome density in transgenic lines carrying *35S::BcCPR5*$^{W7\text{-}2(ATG1)}$. Data are presented as mean ± SD (one-way ANOVA with Tukey's HSD test; different lowercase letters indicate significant differences at $p < 0.05$). (H) Phenotypic comparison of transgenic plants of different genotypes. (a) Whole-plant morphology; (b) close-up views of leaf trichomes; (c) stereomicroscopy images of leaf trichomes; (d) light microscopy images of leaf trichomes; (e) scanning electron microscopy (SEM) images of leaf trichomes; (f) higher-magnification SEM images of leaf trichomes.
