# Supplementary material for: A transposon insertion in the promoter of BcCPR5 impairs trichome formation in wucai (Brassica campestris L.)
Source: Hortic Res. 2026 May 8;13(10):uhag184. doi: 10.1093/hr/uhag184 (PMC13610597; doi:10.1093/hr/uhag184)
Supplement: Supplementary file 1 [file web_material_uhag184.zip › FigureS6.pdf]

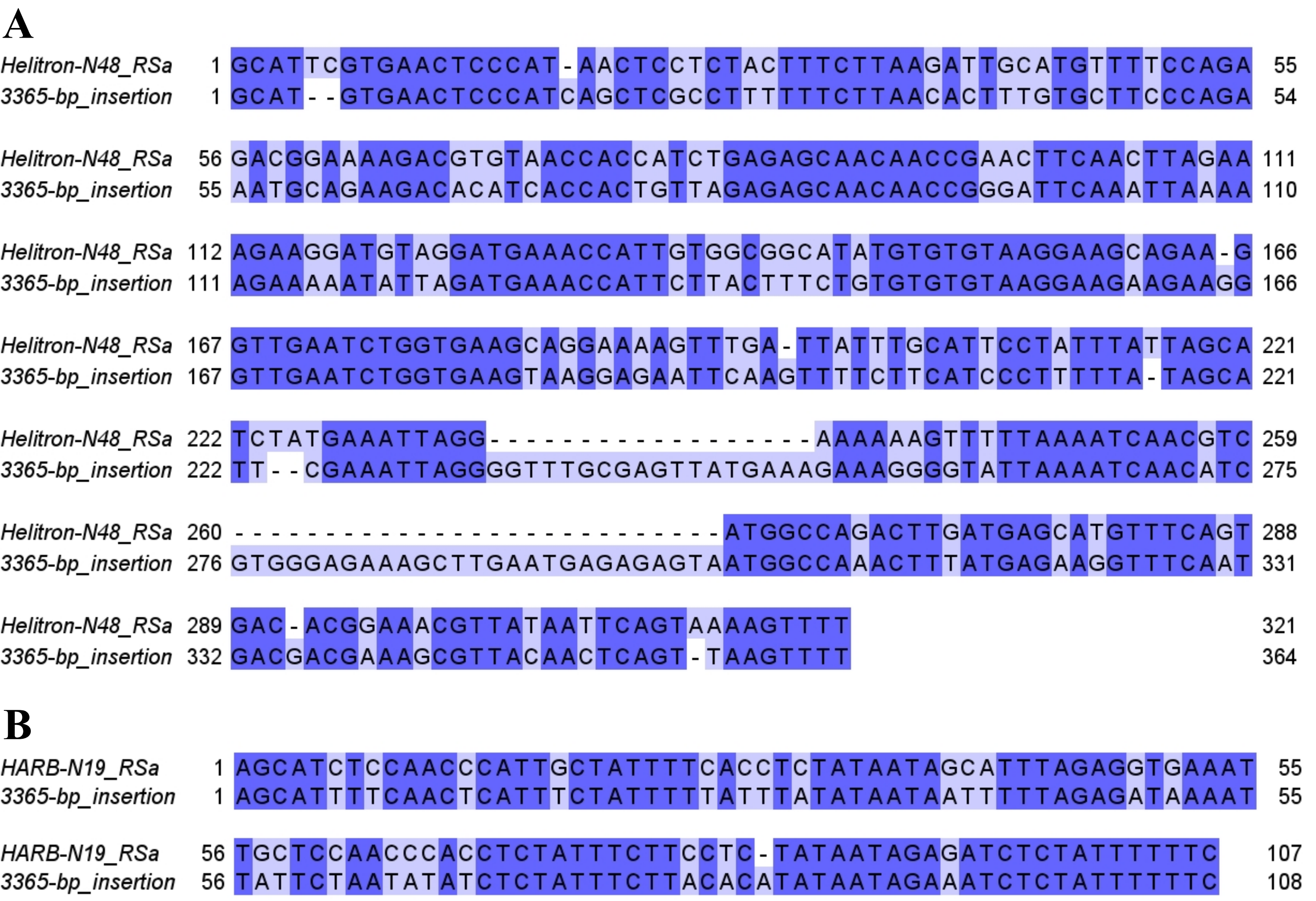

Figure S6. Identification of transposable elements in the W7-2 specific promoter insertion sequence. (A). The Helitron-N48_RSa transposable element is located in the promoter region (-2479 to -2852) of the *BcCPR5*$^{W7-2}$; (B). The HARB-N19_RSa transposable element is located in the promoter region (-337 to -444) of the *BcCPR5*$^{W7-2}$.
