# Supplementary material for: A transposon insertion in the promoter of BcCPR5 impairs trichome formation in wucai (Brassica campestris L.)
Source: Hortic Res. 2026 May 8;13(10):uhag184. doi: 10.1093/hr/uhag184 (PMC13610597; doi:10.1093/hr/uhag184)
Supplement: Supplementary file 1 [file web_material_uhag184.zip › FigureS7.pdf]

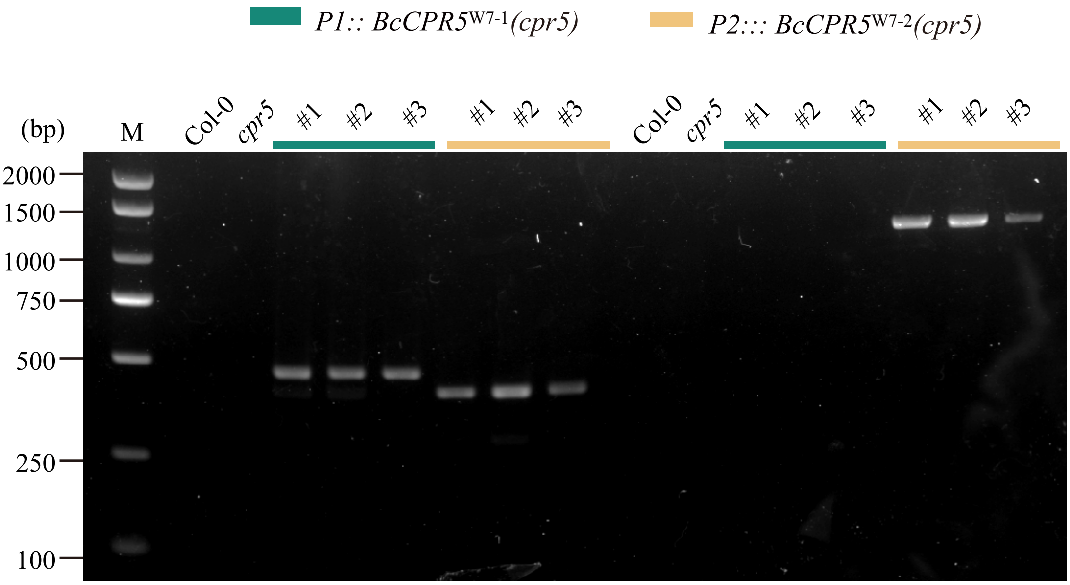

Figure S7. PCR-based genotyping confirmation of transgenic plants carrying *P1:: BcCPR5*$^{W7-1}$ and *P2:: BcCPR5*$^{W7-2}$.
