# Supplementary material for: A transposon insertion in the promoter of BcCPR5 impairs trichome formation in wucai (Brassica campestris L.)
Source: Hortic Res. 2026 May 8;13(10):uhag184. doi: 10.1093/hr/uhag184 (PMC13610597; doi:10.1093/hr/uhag184)
Supplement: Supplementary file 1 [file web_material_uhag184.zip › FigureS8.pdf]

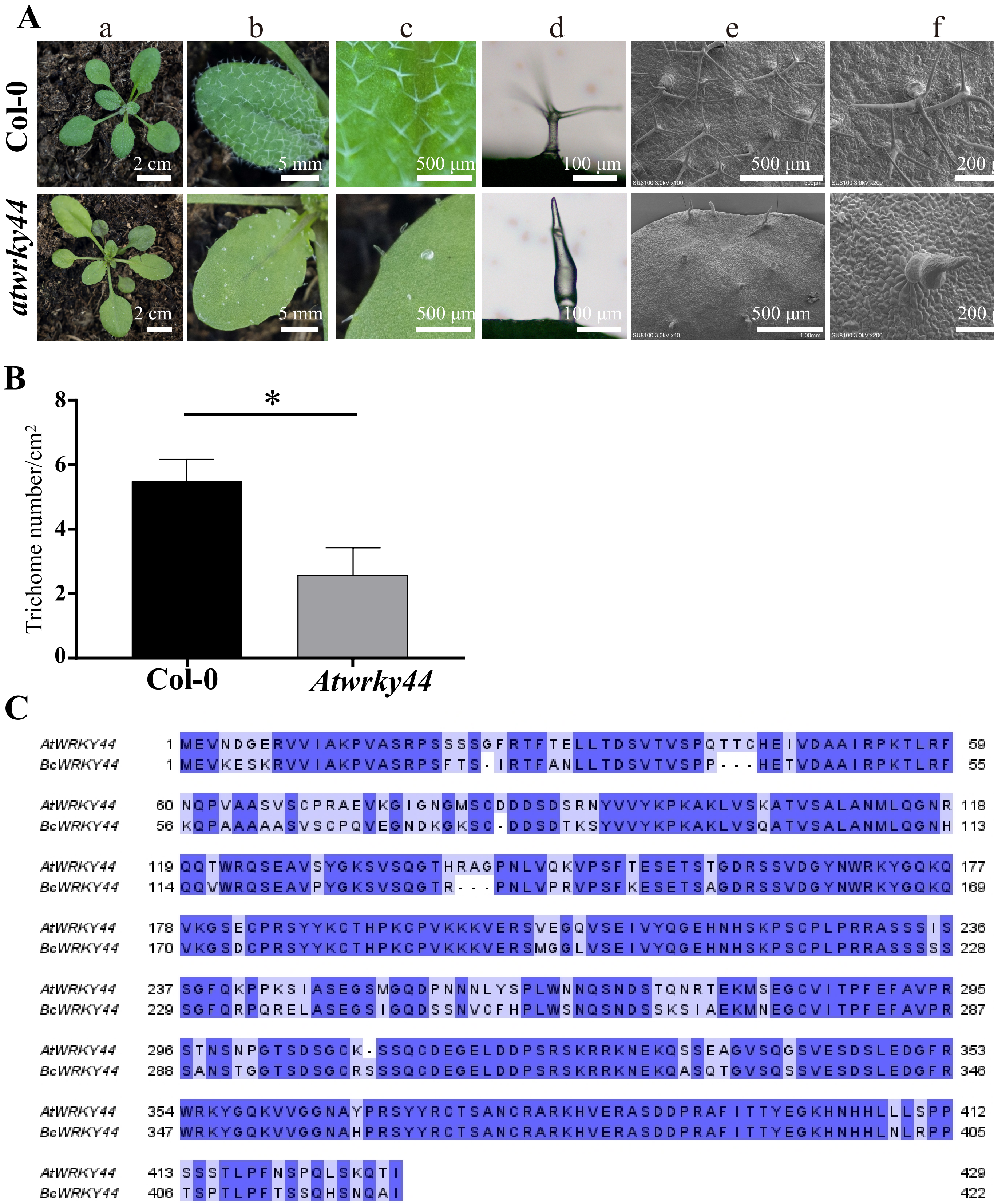

Figure S8. Phenotypic characterization of the *wrky44* mutant and sequence conservation of WRKY44. (A) Phenotypic comparison between Col-0 and *wrky44* mutant. (a) Whole-plant morphology; (b) close-up views of leaf trichomes; (c) stereomicroscopy images of leaf trichomes; (d) light microscopy images of leaf trichomes; (e) SEM images of leaf trichomes; (f) higher-magnification SEM images of leaf trichomes. (B) Quantitative analysis of trichome density in wild-type and *wrky44* mutant. Data are presented as mean ± SD (Student's t-test, *p < 0.05). (C) Amino acid sequence alignment of BcWRKY44 and AtWRKY44.
