# Supplementary material for: A transposon insertion in the promoter of BcCPR5 impairs trichome formation in wucai (Brassica campestris L.)
Source: Hortic Res. 2026 May 8;13(10):uhag184. doi: 10.1093/hr/uhag184 (PMC13610597; doi:10.1093/hr/uhag184)
Supplement: Supplementary file 1 [file web_material_uhag184.zip › FigureS9.pdf]

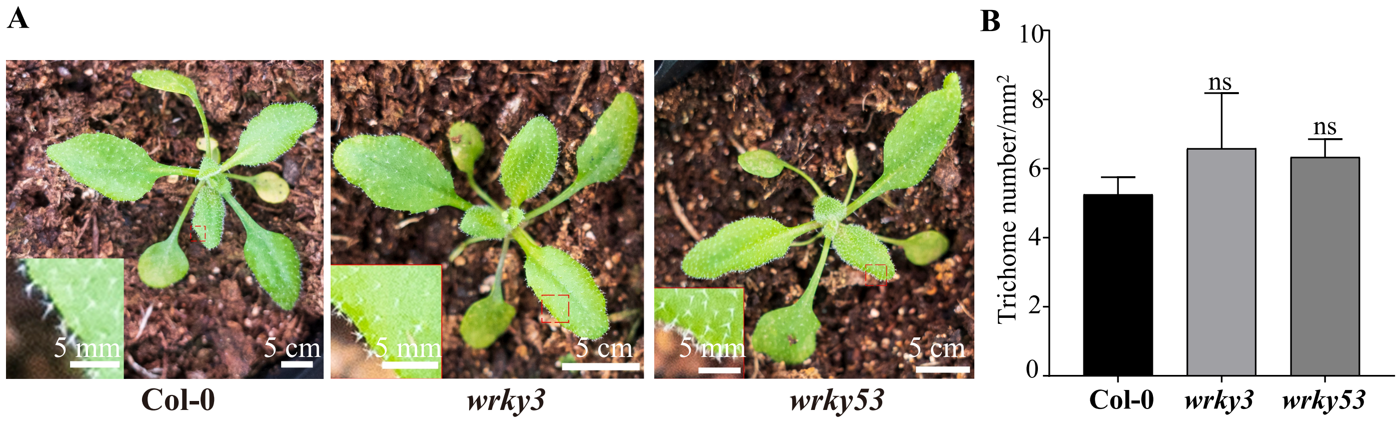


**Figure S9** Phenotypic analysis of *wrky3* and *wrky53* mutants. (A) Phenotypic comparison between Col-0 and *wrky3* and *wrky53* mutants. (B) Quantitative analysis of trichome density in wild-type and *wrky3* and *wrky53* mutants. Data are presented as mean ± SD (Student's t-test; ns, not significant).
